# DNA methylation and associated gene expression in blood prior to lung cancer diagnosis in the Norwegian Women and Cancer cohort

**DOI:** 10.1038/s41598-018-34334-6

**Published:** 2018-11-13

**Authors:** Torkjel Manning Sandanger, Therese Haugdahl Nøst, Florence Guida, Charlotta Rylander, Gianluca Campanella, David C. Muller, Jenny van Dongen, Dorret I. Boomsma, Mattias Johansson, Paolo Vineis, Roel Vermeulen, Eiliv Lund, Marc Chadeau-Hyam

**Affiliations:** 1 Department of Community Medicine, Faculty of Health Sciences, UiT – The Arctic University of Norway, Tromsø, Norway; 2 MRC/PHE Centre for Environmental Health, Department of Epidemiology and Biostatistics, Imperial College London, London, United Kingdom; 3 Genetic Epidemiology Group, International Agency for Research on Cancer, Lyon, France; 4 Netherlands Twin Register, Vrije Universiteit, Department of Biological Psychology, Amsterdam, The Netherlands; 5 Italian Institute for Genomic Medicine (IIGM), Turin, Italy; 6 Institute for Risk Assessment Sciences (IRAS), Division of Environmental Epidemiology, Utrecht University, Utrecht, The Netherlands

## Abstract

The majority of lung cancer is caused by tobacco smoking, and lung cancer-relevant epigenetic markers have been identified in relation to smoking exposure. Still, smoking-related markers appear to mediate little of the effect of smoking on lung cancer. Thus in order to identify disease-relevant markers and enhance our understanding of pathways, a wide search is warranted. Through an epigenome-wide search within a case-control study (131 cases, 129 controls) nested in a Norwegian prospective cohort of women, we found 25 CpG sites associated with lung cancer. Twenty-three were classified as associated with smoking (LC-AwS), and two were classified as unassociated with smoking (LC-non-AwS), as they remained associated with lung cancer after stringent adjustment for smoking exposure using the comprehensive smoking index (CSI): cg10151248 (*PC*, CSI-adjusted odds ratio (OR) = 0.34 [0.23–0.52] per standard deviation change in methylation) and cg13482620 (*B3GNTL1*, CSI-adjusted OR = 0.33 [0.22–0.50]). Analysis among never smokers and a cohort of smoking-discordant twins confirmed the classification of the two LC-non-AwS CpG sites. Gene expression profiles demonstrated that the LC-AwS CpG sites had different enriched pathways than LC-non-AwS sites. In conclusion, using blood-derived DNA methylation and gene expression profiles from a prospective lung cancer case-control study in women, we identified 25 CpG lung cancer markers prior to diagnosis, two of which were LC-non-AwS markers and related to distinct pathways.

## Introduction

Lung cancer is the leading cause of cancer death worldwide, causing as many deaths as the next four most deadly cancers combined (breast, prostate, colon, and pancreas), and the incidence of lung cancer is projected to double by 2050^1^.

Recent advances in molecular biology, and the emergence of cost efficient OMICs data has contributed new insight into mechanisms involved in lung carcinogenesis. For instance, several epigenome-wide association studies have identified methylation changes that are associated with lung cancer risk^2–8^, using mainly tumor tissue collected at the time of diagnosis, but also blood in prevalent cases^9^. Other studies focusing on established lung carcinogens such as smoking used peripheral blood and identified several differentially methylated CpG sites^10–13^. Of these, a recent meta-analysis including 15,907 participants identified 2,623 differentially methylated CpG sites in relation to smoking status^14^.

Although smoking is an established causal risk factor responsible for a vast proportion of lung cancer incidence, identified smoking-related CpG sites have been shown to mediate some or little of the effects of smoking on lung cancer^6,15^. Further, the most common histological subtype of lung cancer in never smokers is adenocarcinoma^16^, which might originate from specific, and as yet unknown, molecular mechanisms different from those involved in smoking-induced lung cancer^17^. It is therefore of interest to identify lung cancer biomarkers that are unrelated to smoking exposure to (i) gain better understanding of the etiology of lung cancer and (ii) to investigate whether biological pathways affected by smoking-induced changes in DNA-methylation are similar to those affected by differential methylation at CpG sites that are not associated with smoking exposure. To-date, very few studies have investigated methylation markers of lung cancer risk that are not associated with such exposure^8,18^.

In the current study, we used full-resolution DNA methylation profiles from prospectively collected blood samples to identify methylation alterations in relation to future lung cancer diagnosis and assess the relationship between these markers and smoking exposure. Specifically, we adopted a stringent adjustment strategy to identify disease-related methylation changes at CpG sites that are not associated with smoking exposure and compare them to changes in methylation level at disease-related CpG sites that are also associated with smoking exposure. Finally, we exploited gene expression data measured in the same individuals to aid functional interpretation by exploring biological pathways of gene expression profiles affected by methylation changes at all lung cancer-related CpG sites.

## Materials and Methods

### Participants

Our study population included women from a lung cancer case–control study nested in the post genome cohort (N ~ 50 000) within the Norwegian Women and Cancer Study (NOWAC)^19–21^. All participating women were cancer-free at recruitment (1991–2006) and at time of blood sampling (2003–2006). Linkage to the national cancer registry identified 134 incident lung cancer cases. Cases were diagnosed between 2004 and 2011, and for each case, one control was matched on time since blood sampling and birth year. All participants gave written informed consent and the study was approved by the Regional Committee for Medical and Health Research Ethics and the Norwegian Data Inspectorate. We confirm that all methods employed in the study were performed in accordance with the relevant guidelines and regulations.

We used methylation data from the Netherlands Twin Register (NTR) for replication. Subjects in the NTR biobank study were recruited between 2004 and 2011^22,23^. The study included 769 monozygotic (MZ) and 424 dizygotic (DZ) twin pairs. A blood sample was collected at inclusion and we included in the present study 125 MZ and 146 DZ adult twin pairs who were discordant with respect to their smoking status at time of blood sampling. We included pairs in which one twin never smoked and the other twin was a current smoker (*N* = 53 MZ, and 77 DZ), and pairs including one never smoker and one former smoker (*N* = 72 MZ, and 69 DZ).

### DNA methylation and gene expression microarray data

Genome-wide DNA methylation profiles from bisulphite-converted, hybridized genomic DNA from buffy coat samples were generated using Illumina Infinium HumanMethylation450 Bead-Chips following a protocol described previously for both NOWAC^24^ and NTR^22,25^ samples. DNA methylation levels at each locus were expressed as the ratio of intensities arising from methylated cytosines over total intensities. For NOWAC, sample preparation and data pre-processing were performed as described elsewhere^24^. In brief, probes (i) on sex chromosomes, (ii) reported to be cross-reactive^26^ and (iii) for which methylation levels were measured in <20% of the samples were excluded. Five samples did not pass quality controls and three subjects were excluded due to >95% missing in DNA methylation results. The final analysis included 428,629 probes targeting autosomal CpG loci in 260 women (131 cases and 129 controls). In NTR data, sample- and probe-level quality checks and data pre-processing were performed as described in detail previously^25^ and only CpG sites identified in the NOWAC discovery data were interrogated.

For 248 of the 260 women from the NOWAC study with DNA methylation data, gene expression profiles were also available and were generated at the Norwegian University of Science and Technology. Total RNA was isolated using established protocols^27^ and microarray analyses were performed using the IlluminaHuman HT-12 expression Bead-Chips. Microarray data were quality-checked and pre-processed as previously described^28^. Original probe values were background-corrected and probes reported to have poor quality from Illumina or detected in <95% of samples were filtered out. Only transcripts on autosomal chromosomes were included in the analysis. The final gene expression data set included 18,955 transcripts assayed in 248 individuals.

### Statistical models

We investigated the relationship between future lung cancer status and methylation levels using unconditional logistic regression models. As already described^29^, we corrected for technically-induced variation in methylation and gene expression data by fitting a preliminary linear mixed model including technical covariates (chip ID and position on the chip for methylation data and date of mRNA isolation and date of complementary RNA generation for gene expression data) as random intercepts, and, to account for the case control matching we adjusted (fixed effects) our models for the two matching criteria: age at blood collection and sample storage time.

Methylation and gene expression levels used in the downstream analyses were represented by the residuals from these mixed models. Multiple testing was accounted for by using a Bonferroni correction ensuring a family-wise error rate below 5% (corresponding per test significance level was set to 1.16e-07). We report as effect size estimates the odds ratios (OR) for one standard deviation change in the methylation levels.

We further adjusted the logistic regression models for blood cell composition, estimated according to the methods proposed by Houseman^30,31^. We specifically adjusted for estimated proportions of leukocytes (excluding natural killer cells and eosinophil granulocytes).

Lung cancer-related CpG sites that are not associated with smoking exposure (LC-non-AwS) were defined as those (i) found significantly associated to lung cancer in the main logistic model, and (ii) remaining associated to lung cancer upon adjustment for smoking. Conversely, lung cancer-related CpG sites that are associated with smoking exposure (LC-AwS) are defined as those losing statistical significance upon adjustment for smoking exposure. Confirmation of their lack of association to smoking exposure was sought in never smokers and an independent study including smoking-discordant twins. We investigated three measures of smoking exposure: smoking status, pack-years, and the comprehensive smoking index (CSI)^32^. CSI scores (Table S1) were obtained using duration of smoking (dur; years), intensity (int; average number of cigarettes per day during years of smoking), and time since smoking cessation (tsc; years) and fitting the following model to our data:

*X*_2_ = (1 − 0.5^dur∗/τ^)(0.5^tsc∗/τ^) ln(int + 1), where τ is the estimated half-life parameter, and δ is an estimated lag time parameter describing tsc and total duration as follows: tsc∗ = max(tsc − δ, 0) and dur∗ = max(dur + tsc − δ) − tsc∗.

To further assess possible relations between methylation levels at disease-related sites and smoking exposure, we used the methylation data of the NTR study and ran paired Student’s T-test analyses comparing the mean methylation differences within pairs of MZ and DZ smoking discordant twins. Paired T-tests were performed on residual methylation levels, which were obtained by adjusting the methylation levels (beta-values) for sex, age at blood sampling, measured cell counts (percentage of monocytes, eosinophils, and neutrophils), and technical covariates: array row and sample plate.

We ran a series of sensitivity analyses that included conditional logistic regressions for the (N = 128) case-control complete pairs. Further sensitivity analyses were restricted to (i) cases from each of the main histological subtypes separately (adenocarcinomas (N = 64), small cell and squamous (N = 43), others (N = 24))^33^, (ii) cases diagnosed before or after the median time elapsed from blood collection to diagnosis (4.2 years), (iii) cases that were current (N = 81), former (N = 36), or never (N = 14) smokers, separately. In these stratified analyses subsets of cases were compared to all healthy controls (N = 37, 35, 57 in current, former and never smokers) included in the study and, because case-control pairs were broken, we used unconditional logistic regression models as defined for the main analysis.

In order to ensure a wide explorative search of (N = *n*_1_) LC-non-AwS markers, which are likely weaker and less numerous than the (N = *n*_2_) LC-AwS markers, we complemented our list of *n*_1_ LC-non-AwS markers by defining a ‘second order’ set of (N = *n*_1_′) LC-non-AwS CpG sites as defined by those associated to a least one of the *n*_1_ LC-non-AwS markers, but not directly to disease status. These were identified by regressing the methylation levels of the *n*_1_ LC-non-AwS CpG sites against the (428,629 − *n*_1_) remaining CpG sites. As before, we used Bonferroni corrected per-test significance level here defined as 0.05/(*n*_1_x(428,629 − *n*_1_)).

In order to help functional interpretation of the resulting epigenetic alterations, gene expression data measured in the same individuals were linked to the DNA methylation levels of the identified markers. Specifically, we ran linear regression models assessing the association between the 18,955 assayed transcripts and (i) each of the first and second order (*n*_1_ + *n*_1_′) LC-non-AwS CpG sites and (ii) each of the *n*_2_ LC-AwS CpG sites. Statistical significance of each CpG-transcript pair was evaluated adopting a Bonferroni corrected per-test significance level (0.05/((*n*_1_ + *n*_1_′) × 18,955), and 0.05/(*n*_2_ × 18,955), respectively). The transcripts involved in any significant CpG-transcript pair were subsequently included in overrepresentation analyses based on hypergeometric tests setting a nominal *p*-value of 0.05 using the ‘enrichGO’ function of the Bioconductor ‘clusterProfiler‘ package^34^. All statistical analyses were performed using R (ver. 3.1.2, Foundation for Statistical Computing, Vienna, Austria).

## Results

### Sample description and overall lung cancer risk

Baseline characteristics of the NOWAC women and NTR study populations are summarized in Table 1. As expected, NOWAC cases were more commonly current smokers at blood sampling (62%) than controls (29%) (Table S1). Logistic regression models demonstrated elevated lung cancer risk in former smokers (OR = 4.07 (95% CI: 1.97–8.79), and in current smokers (OR = 8.46 (95% CI: 4.31–17.53)) as compared to never smokers. The model including CSI score alone indicated an OR of 3.66 (95% CI: 2.53–5.42) for one unit increase in CSI values and the model provided the better fit compared to other smoking metrics (including smoking status and pack years, AIC results not shown). Estimated cell type proportions were similar in cases and controls, except for the natural killer cells, which were underrepresented in cases and among current smokers (Table S2).

**Table 1:** Characteristics of the NOWAC (women only) and the NTR populations.

Variable	NOWAC study				NTR study			
	Cases *N* = 131		Controls *N* = 129		Monozygotic *N* = 250 (125 pairs)		Dizygotic *N* = 292 (146 pairs)	
	Mean	SD	Mean	SD	Mean	SD	Mean	SD
Age at sample	56.55	4.01	56.57	3.98	37.77	11.91	33.24	8.18
Age at diagnosis	60.5	4.12						
Time to diagnosis	3.88	1.99						
Pack-years	20.46	13.3	8.62	11.18				
CSI	1.3	0.65	0.63	0.71				
	***N***	**%**	***N***	**%**	***N***	**%**	***N***	**%**
**Histological subtypes**								
Adenocarcinomas	64	48.9						
Small cell carcinomas	25	19.1						
Squamous cell carcinomas	18	13.7						
Others	24	18.3						
**Smoking status**								
Current	81	61.8	37	28.68	53	21.2	77	26.4
Former	36	27.5	35	27.13	72	28.8	69	23.6
Never	14	10.7	57	44.19	125	50	146	50

### Differentially methylated CpG sites associated to lung cancer risk

**Figure 1:**
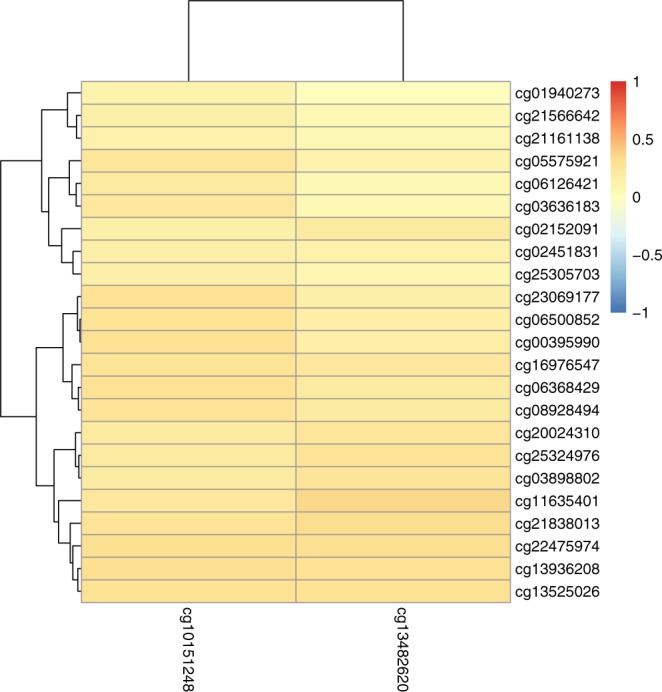
Heatmap of the correlation between the two CpGs un-associated with smoking (LC-non-AwS) and the 23 CpGs associated with smoking (LC-AwS). Figure note: The correlation strength is represented by color as indicated in the bar to the right.

We identified 25 CpG sites at which lower methylation levels were associated to higher lung cancer risk (Tables 2 and S3; boxplots of the methylation according to case/control status in Figure S1 and volcano plot in Figure S2). After adjustment for smoking, *n*_2_ = 23 of these sites were classified as LC-AwS markers, as their associations lost statistical significance (Table 2). Among the different smoking metrics considered, CSI appeared to provide the most stringent adjustment as depicted by flattened *p*-value distribution (Figure S3, estimates for the covariates adjusted for are presented in Table S4). Only *n*_1_ = 2 CpGs remained associated with lung cancer risk after controlling for CSI and were classified as LC-non-AwS markers (Table 2): cg10151248; *PC* (OR = 0.34) and cg13482620; *B3GNTL*1 (OR = 0.33). These two LC-non-AwS CpGs were also significantly associated with lung cancer after further adjustment for blood cell composition (Table S5). The correlations between the *n*_*1*_ = 2 LC-non-AwS CpG sites and the *n*_2_ = 23 LC-AwS sites were moderate (Fig. 1). Conversely, we observed stronger block correlations within the LC-AwS sites, and in particular a subset of eight CpG sites (Figure S4). Results in figures and tables are presented separately for LC-AwS and LC-non-AwS sites.

**Table 2:** 25 Bonferroni significant CpG sites differentially methylated in cases as compared to controls (N = 131 cases, 129 controls) that were un-associated with smoking (LC-non-AwS), or associated with smoking (LC-AwS).

Probe ID	Gene name	Chromosome	Unadjusted model			Adjusted model - smoking status			Adjusted model - pack-years			Adjusted model - CSI		
			OR	95% CI	*p*-value	OR	95% CI	*p*-value	OR	95% CI	*p*-value	OR	95% CI	*p*-value
***LC-Non-AwS CpG sites***														
cg10151248	*PC*	11	0.36	0.25–0.51	**1.2E-08**	0.36	0.25–0.52	**7.2E-08**	0.35	0.24–0.51	**5.0E-08**	0.34	0.23–0.5	**7.1E-08**
cg13482620	*B3GNTL1*	17	0.41	0.3–0.57	**8.5E-08**	0.39	0.27–0.55	2.1E-07	0.33	0.22–0.5	**6.7E-08**	0.33	0.22–0.5	**8.4E-08**
***LC-AwS CpG sites***														
cg05575921	*AHRR*	5	0.37	0.27–0.49	**2.9E-11**	0.38	0.23–0.64	2.5E-04	0.55	0.37–0.81	2.2E-03	0.56	0.35–0.9	1.6E-02
cg03636183	*F2RL3*	19	0.38	0.28–0.52	**2.5E-10**	0.49	0.32–0.75	8.5E-04	0.58	0.4–0.83	3.2E-03	0.58	0.38–0.89	1.2E-02
cg06126421	*NA*	6	0.38	0.28–0.52	**8.6E-10**	0.54	0.36–0.8	2.1E-03	0.59	0.41–0.85	4.7E-03	0.63	0.42–0.94	2.5E-02
cg21566642	*NA*	2	0.42	0.31–0.56	**2.5E-09**	0.60	0.38–0.94	2.7E-02	0.67	0.46–0.98	3.7E-02	0.69	0.43–1.11	1.3E-01
cg02152091	*NA*	8	0.39	0.29–0.53	**3.7E-09**	0.39	0.28–0.55	**2.8E-08**	0.40	0.28–0.55	**6.5E-08**	0.43	0.31–0.6	5.3E-07
cg03898802	*DOPEY2*	21	0.39	0.28–0.53	**6.8E-09**	0.41	0.29–0.58	2.6E-07	0.41	0.29–0.58	4.4E-07	0.42	0.29–0.59	8.7E-07
cg06500852	*NA*	2	0.40	0.29–0.55	**7.8E-09**	0.43	0.31–0.6	5.7E-07	0.45	0.33–0.63	2.5E-06	0.46	0.33–0.64	4.9E-06
cg20024310	*NA*	7	0.37	0.26–0.52	**1.7E-08**	0.35	0.24–0.52	**9.2E-08**	0.36	0.24–0.53	2.7E-07	0.36	0.24–0.54	6.0E-07
cg06368429	*KPNA7*	7	0.39	0.28–0.54	**1.9E-08**	0.44	0.31–0.61	2.0E-06	0.41	0.28–0.58	5.6E-07	0.43	0.3–0.62	4.1E-06
cg02451831	*KIAA0087*	7	0.41	0.3–0.56	**1.9E-08**	0.50	0.35–0.7	8.4E-05	0.53	0.38–0.74	1.9E-04	0.54	0.39–0.76	4.0E-04
cg13936208	*NA*	12	0.38	0.27–0.53	**2.1E-08**	0.38	0.26–0.55	3.7E-07	0.42	0.29–0.6	2.1E-06	0.41	0.29–0.6	2.8E-06
cg13525026	*MYO15A*	17	0.43	0.32–0.58	**2.3E-08**	0.48	0.35–0.66	5.0E-06	0.48	0.35–0.66	7.8E-06	0.48	0.34–0.66	9.6E-06
cg08928494	*CA5A*	16	0.39	0.28–0.55	**2.7E-08**	0.41	0.29–0.58	5.8E-07	0.39	0.27–0.57	4.7E-07	0.40	0.28–0.58	8.6E-07
cg25305703	*NA*	8	0.43	0.32–0.58	**3.2E-08**	0.56	0.4–0.78	6.0E-04	0.57	0.41–0.79	6.4E-04	0.61	0.44–0.85	3.3E-03
cg00395990	*PDZD3*	11	0.42	0.31–0.57	**3.7E-08**	0.46	0.34–0.64	3.3E-06	0.48	0.34–0.66	1.2E-05	0.50	0.36–0.69	3.8E-05
cg25324976	*CSHL1*	17	0.43	0.31–0.58	**6.0E-08**	0.45	0.32–0.63	2.7E-06	0.45	0.32–0.63	5.6E-06	0.48	0.34–0.67	2.1E-05
cg01940273	*NA*	2	0.46	0.35–0.61	**6.1E-08**	0.67	0.44–1	5.1E-02	0.73	0.51–1.03	7.1E-02	0.79	0.53–1.17	2.4E-01
cg11635401	*MYO9B*	19	0.43	0.31–0.58	**9.1E-08**	0.40	0.28–0.57	3.0E-07	0.40	0.28–0.57	2.5E-07	0.41	0.29–0.58	5.7E-07
cg22475974	*NA*	4	0.44	0.33–0.6	**9.2E-08**	0.47	0.34–0.64	2.2E-06	0.48	0.35–0.65	3.6E-06	0.50	0.37–0.69	2.1E-05
cg21838013	*CRTAM*	11	0.41	0.3–0.57	**1.0E-07**	0.44	0.31–0.62	2.1E-06	0.42	0.3–0.6	1.3E-06	0.45	0.32–0.64	6.3E-06
cg23069177	*OCA2*	15	0.43	0.31–0.58	**1.0E-07**	0.43	0.3–0.6	1.7E-06	0.43	0.31–0.61	2.3E-06	0.41	0.29–0.59	1.4E-06
cg21161138	*AHRR*	5	0.45	0.34–0.61	**1.0E-07**	0.66	0.45–0.95	2.7E-02	0.70	0.5–0.97	3.3E-02	0.77	0.54–1.11	1.6E-01
cg16976547	*FES*	15	0.42	0.31–0.58	**1.1E-07**	0.44	0.31–0.62	3.2E-06	0.45	0.32–0.64	6.4E-06	0.46	0.33–0.66	1.6E-05

OR, odds ratio; CI, confidence interval. Regression models include residual DNA methylation levels (DNA methylation adjusted for technical covariates and matching variables; see Method section) as an independent variable. Results are presented for the unadjusted unconditional logistic regression and the same model adjusted for three selected smoking metrics (smoking status as categories never/former/current, pack-years, and comprehensive smoking index (CSI)). Model results for unconditional logistic regressions adjusted for WBCs and CSI + WBCs are presented in Table S4. Bolded *p*-values are significant according to the Bonferroni threshold.

The two LC-non-AwS CpG sites were also associated with lung cancer risk in never smokers (OR = 0.36 (95% CI: 0.17–0.77) and OR = 0.31 (95% CI: 0.14–0.67) for cg10151248-*PC* and cg13482620-*B3GNTL*1, respectively). Estimates were consistent in current smokers for cg10151248-*PC* and cg13482620-*B3GNTL1* (OR = 0.32 (95% CI: 0.19–0.57) and OR = 0.33 (95% CI: 0.18–0.61)), respectively) but slightly weaker in former smokers (OR = 0.43 (95% CI: 0.23–0.82) and 0.50 (95% CI: 0.30–0.85)). The stratified analysis showed that 10 CpG sites among the 23 LC-AwS CpG sites were significantly associated to lung cancer status in never smokers (Table S6). The methylation levels of the two most strongly associated LC-AwS CpG sites: cg05575921-*AHRR* and cg03636183-*F*2*RL3*, were not associated with lung cancer risk in never smokers (OR = 0.27 (95% CI: 0.03–2.14) and 1.22 (95% CI: 0.32–4.68), respectively.

Additional stratification on histological subtypes provided consistent OR estimates for cg10151248-*PC* across histological subtypes (Table S7; range: 0.36–0.39), and stronger effects of methylation levels were estimated in cases with shorter time to diagnosis (0.33 vs 0.41 for short and long time to diagnosis, respectively). For cg13482620-*B3GNTL1*, effect size estimates were consistent in both time to diagnosis classes, but the OR was lower in adenocarcinoma cases (OR = 0.35) than in ‘all other subtypes’ and ‘squamous and small cell’ cases (OR >0.49). Corresponding stratified analyses for LC-AwS CpGs are also presented in Table S7.

Using conditional logistic regressions unadjusted for smoking exposure, as a sensitivity analysis, only two of the *n*_*1*_ + *n*_*2*_ = 25 candidate CpG sites reached Bonferroni significance level (cg05575921 and cg06126421, *p*-values 3.99e^−08^ and 6.68e^−08^, respectively).

When comparing mean methylation levels for the 25 candidate CpG sites within pairs of smoking-discordant twins (MZ or all), we found no differences between never smokers and ever/current smokers for the two LC-non-AwS markers (Table 3). Comparison of the mean methylation levels at the LC-AwS CpG sites, showed significant differences at eight CpG sites while comparing smokers (current or ever) to never smokers. When restricting these comparisons to MZ twin pairs, six and eight CpG sites were significantly different in never to current and never to ever comparisons, respectively (Table 3).

**Table 3:** Difference in methylation in twins discordant according to smoking status in the NTR study for the CpG sites associated with lung cancer identified as un-associated with smoking (LC-non-AwS), or associated with smoking (LC-AwS) in the NOWAC study.

CpG site	All twins						Monozygotic twins					
	Never vs. Current			Never vs. Ever			Never vs. Current			Never vs. Ever		
	Mean diff.	95% CI	*p*-value	Mean diff.	95% CI	*p*-value	Mean diff.	95% CI	*p*-value	Mean diff.	95% CI	*p*-value
***LC-Non-AwS CpG sites***												
cg10151248	−0.001	−0.003–0.001	2.7E-01	0.000	−0.002–0.001	5.1E-01	−0.002	−0.005–0	8.3E-02	0.000	−0.002–0.001	6.4E-01
cg13482620	−0.002	−0.005–0.002	3.7E-01	−0.002	−0.004–0.001	1.7E-01	−0.002	−0.008–0.004	5.6E-01	−0.002	−0.005–0.002	3.1E-01
***LC-AwS CpG sites***												
cg05575921	0.139	0.118–0.159	**7.8E-26**	0.086	0.073–0.099	**4.5E-31**	0.132	0.1–0.164	**3.0E-11**	0.073	0.055–0.09	**2.8E-13**
cg03636183	0.065	0.053–0.077	**1.4E-20**	0.045	0.037–0.052	**6.2E-27**	0.062	0.045–0.08	**2.9E-09**	0.039	0.029–0.049	**4.7E-13**
cg06126421	0.056	0.044–0.067	**1.6E-16**	0.042	0.035–0.049	**9.4E-27**	0.050	0.033–0.067	**2.9E-07**	0.034	0.025–0.042	**1.3E-11**
cg21566642	0.092	0.077–0.107	**7.5E-23**	0.067	0.058–0.077	**7.6E-34**	0.092	0.069–0.115	**1.2E-10**	0.059	0.047–0.072	**1.8E-15**
cg02152091	−0.003	−0.007–0.002	2.0E-01	−0.003	−0.006–0	4.8E-02	0.000	−0.007–0.007	9.9E-01	−0.003	−0.007–0.001	1.6E-01
cg03898802	−0.002	−0.005–0.001	1.5E-01	−0.001	−0.003–0	1.6E-01	−0.002	−0.006–0.003	4.4E-01	0.000	−0.002–0.003	8.5E-01
cg06500852	−0.002	−0.004–0.001	1.5E-01	−0.001	−0.002–0.001	5.3E-01	−0.004	−0.008–0.001	2.6E-02	−0.002	−0.004–0.001	1.8E-01
cg20024310	−0.006	−0.011–0	6.5E-02	−0.003	−0.007–0.001	1.7E-01	−0.004	−0.012–0.005	4.1E-01	0.003	−0.003–0.009	2.7E-01
cg06368429	−0.006	−0.013–0.001	9.5E-02	−0.003	−0.007–0.001	1.6E-01	−0.008	−0.019–0.003	1.5E-01	−0.002	−0.008–0.004	5.1E-01
cg02451831	0.014	0.009–0.02	**3.0E-06**	0.010	0.006–0.013	**1.8E-07**	0.013	0.005–0.021	2.9E-03	0.008	0.003–0.013	**1.2E-03**
cg13936208	−0.004	−0.008–0	4.5E-02	−0.003	−0.006–0	2.2E-02	−0.003	−0.008–0.003	2.8E-01	−0.001	−0.005–0.002	4.8E-01
cg13525026	−0.003	−0.006–0.001	1.2E-01	−0.001	−0.003–0.001	2.9E-01	−0.007	−0.012–0.002	6.0E-03	−0.003	−0.007–0	5.0E-02
cg25305703	0.021	0.012–0.03	**8.9E-06**	0.015	0.01–0.021	**3.1E-07**	0.020	0.006–0.034	5.3E-03	0.013	0.005–0.021	**1.2E-03**
cg00395990	−0.002	−0.006–0.002	3.1E-01	−0.001	−0.003–0.001	3.2E-01	−0.002	−0.007–0.004	5.2E-01	0.000	−0.003–0.003	8.5E-01
cg01940273	0.059	0.049–0.07	**2.3E-20**	0.042	0.036–0.049	**5.9E-29**	0.058	0.042–0.074	**1.7E-09**	0.037	0.028–0.046	**2.1E-13**
cg11635401	−0.002	−0.007–0.003	4.5E-01	−0.001	−0.004–0.002	5.5E-01	−0.006	−0.014–0.002	1.5E-01	−0.003	−0.008–0.002	1.9E-01
cg22475974	0.000	−0.002–0.001	6.1E-01	0.000	−0.002–0.001	4.3E-01	−0.002	−0.004–0.001	3.0E-01	0.000	−0.002–0.001	6.5E-01
cg21838013	−0.003	−0.008–0.002	2.1E-01	−0.001	−0.005–0.003	6.0E-01	−0.001	−0.009–0.007	8.5E-01	0.002	−0.003–0.008	4.0E-01
cg23069177	−0.001	−0.005–0.003	6.2E-01	−0.001	−0.003–0.002	5.6E-01	0.000	−0.006–0.006	9.4E-01	−0.001	−0.005–0.003	6.0E-01
cg21161138	0.044	0.036–0.052	**1.1E-20**	0.026	0.021–0.031	**4.3E-20**	0.044	0.031–0.057	**1.5E-08**	0.020	0.013–0.028	**8.3E-07**
cg16976547	0.001	−0.003–0.005	5.8E-01	0.001	−0.001–0.003	5.4E-01	0.003	−0.006–0.012	4.8E-01	0.001	−0.003–0.005	6.6E-01

Diff: difference; Bolded numbers for *p*-values are considered significant using a Bonferroni threshold.

### Functional investigation of the 25 candidate CpG sites

**Figure 2:**
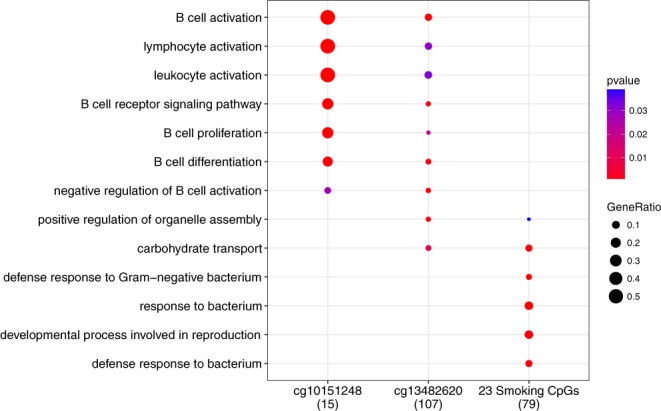
Network visualizations of gene ontology categories in which genes were significantly overrepresented, for the genes associated to the ‘second order’ CpGs un-associated with smoking (CpGs associated with cg10151248-*PC* and cg13482620-*B3GNTL1*) as well as those associated to the 23 CpGs associated with smoking (LC-AwS). Figure note: Biological processes categories are colored according to the significance of the overrepresentation and the gene ratio signifies the number of genes in each list relative to the number of genes in the ontology categories.

No significant association was found linking DNA methylation levels at either LC-non-AwS sites (cg10151248-*PC* and cg13482620-*B3GNTL1)* and the gene expression levels at the 18,955 transcripts assayed (containing one transcript each for *PC* and *B3GNTL1* genes). We identified a total of *n*_1_’ = 1987 ‘second order’ LC-non-AwS CpG sites whose methylation levels were associated to that of at least one of the n_1_ = 2 LC-non-AwS CpG sites, and not directly with disease risk. Of these, 160 and 1,876 were associated with methylation levels of cg10151248-*PC* and cg13482620-*B3GNTL1*, respectively and their pairwise correlation is presented in Figure S5A. When regressing the *n*_1_’ ‘second order’ set of CpG sites against the gene expression levels we identified (i) 19 significant CpG-transcript pairs for cg10151248-*PC* (Table S8), corresponding to 19 unique transcripts and one unique CpG site (Table 4), and (ii) 137 CpG-transcript pairs for cg13482620-*B3GNTL1* (Table S9), including 127 unique transcripts and nine CpG sites (Table 4). The correlations between transcripts associated to the methylation levels of at least one ‘second order’ CpG site are presented in Figure S5B. Overrepresentation analyses of transcripts involved in these significant LC-non-AsW CpG-transcript pairs demonstrated distinct enriched ontology categories relating to immune response, and involving beta cells (Fig. 2, Tables S10 and S11, respectively).

**Table 4:** The number of transcripts associated to the ‘second-order’ CpGs un-associated with smoking (LC-non-AwS) and the CpGs associated with smoking (LC-AwS).

CpG Name	Gene	Chromosome	No. of significant transcripts
***cg10151248- PC associated***			
cg21570493	*NA*	1	19
***cg13482620- B3GNTL1 associated***			
cg03160057	*WDR66*	12	104
cg10115918	*B3GNTL1*	17	11
cg05664421	*ZFYVE28*	4	10
cg13752749	*CRB2*	9	1
cg01676996	*C6orf136*	6	2
cg11654904	*ZNF642*	1	6
cg04909834	*ARHGEF10*	8	1
cg06836020	*LOC440354*	16	1
cg27665823	*DECR2*	16	1
***LC-AwS CpG sites***			
cg06126421	*NA*	6	75
cg05575921	*AHRR*	5	43
cg01940273	*NA*	2	19
cg21566642	*NA*	2	10
cg03636183	*F2RL3*	19	7
cg21161138	*AHRR*	5	7
cg02451831	*KIAA0087*	7	4
cg25305703	*NA*	8	3

For the n_2_ = 23 LC-AwS CpG sites we identified 168 significant CpG-transcript pairs (Tables 4 and S12), corresponding to 100 unique transcripts and eight unique CpG sites. Overrepresentation analyses identified ontology categories distinctly different from those identified above and mostly related to responses to external stressors (Fig. 2 and Table S13).

## Discussion

We combined genome-wide methylation and gene expression profiles from prospective blood samples in the NOWAC study to identify markers of lung cancer risk in Norwegian women and investigated to what extent these associations were driven by exposure to smoking. We identified 25 CpG sites associated with lung cancer risk, of which 23 were classified as LC-AwS, as they lost statistical significance after stringent adjustment for smoking exposure metrics. The two remaining CpG sites (cg10151248-*PC* and cg13482620- *B3GNTL1*) were classified as LC-non-AwS CpG sites, as they remained statistically significant after adjustment for CSI and demonstrated low correlation to the other 23 CpGs. For the majority of markers the case control difference was larger with shorter time to diagnosis.

Of the 23 LC-AwS CpG sites, eight have been acknowledged as epigenetic signatures of cigarette smoking in a recent large meta-analysis of DNA methylation and smoking^14^. Pairwise correlations among the same eight LC-AwS CpG sites were also markedly higher than correlations with the other 15 LC-AwS CpG sites, supporting the evidence of these being linked to smoking. Furthermore, the same eight LC-AwS CpG sites were differentially methylated in smoking discordant twins. For the majority of the 23 LC-AwS markers, the association with risk was also stronger in the smoking related histological subtypes as compared to adenocarcinoma. The evidence to classify the 23 CpG sites as LC-AwS was not equally strong, but was considered sufficient for them to be treated separately as LC-AwS markers in the downstream analyses.

The two LC-non-AwS CpG sites were consistently not associated to smoking exposure in all the analyses performed, which indicates that they are minimally associated with smoking. The association between LC-non-AwS CpG sites and risk was stronger in adenocarcinoma cases compared to the other more smoking-induced histological subtypes^35^, which was not the trend observed in LC-AwS markers. Although hampered by statistical power in stratified analyses of never smokers, the same two LC-non-AwS markers were found to be statistically significant, along with 10 of the LC-AwS CpGs. Comparing pairs of smoking discordant twins revealed no difference in methylation levels at LC-non-AwS CpG sites. Finally, the two LC-non-AwS CpGs were not identified in the large meta-analyses for epigenetic smoking signatures^14^ and we did not identify any single-nucleotide polymorphisms reported in the vicinity of these two sites^36^, hence arguing against possible genetic confounding. Taken together, this supports that the two LC-non-AwS CpG sites, and in particular cg10151248-*PC*, are not associated with smoking and are distinct from the LC-AwS markers.

To enable deeper investigation of the functional role of the methylation changes at the LC-non-AwS CpG sites, we defined ‘second order’ CpG sites as being associated with the methylation levels at any of these two LC-non-AwS CpG sites but not directly with lung cancer risk. None of the 160 CpG sites associated with cg10151248-*PC* were associated with smoking status in a recent large meta-analysis of DNA methylation and smoking status^14^, and 22 of the 1,876 for cg13482620-*B3GNTL1* (1.2%) were reported as LC-AwS markers. On this basis, we explored whether the two non-LC-AwS markers and complemented list of markers less associated with smoking could provide novel pathway information relevant for lung cancer development.

The candidate methylation markers were further investigated by exploring the association between methylation levels at lung cancer related markers and gene expression data available in the same individuals. In order to ensure a comprehensive search for distinguishable pathways the full sets of markers were explored separately for the LC-AwS and LC-non-AwS CpG sites. Because regulation of gene expression through differential methylation obeys complex and multivariate mechanisms and can operate remotely (‘trans’ effects)^37^, all assayed transcripts were investigated. No transcript was directly associated with methylation levels at cg10151248-*PC* and cg13482620-*B3GNTL1* (neither *PC* or *B3GNTL1* transcripts) which may not be surprising as both are highly methylated and show small, although significant, differences between cases and controls. However, we identified associations between methylation levels at the ‘second order’ CpG sites, and transcripts. The significant CpG-transcript pairs for the 160 cg10151248-*PC*-related CpG sites involved 19 transcripts, none of which were AwS markers either in our data or in the large meta-analysis of gene expression data^38^, while 33 of 127 transcripts involved in cg13482620-*B3GNTL1*-related CpG-transcript pairs were identified in the large meta-analysis^38^.

In the exploration of LC-non-AwS markers of lung cancer, which are likely to be more subtle signals than LC-AwS markers, an enriched CpG list was assessed when comparing potential functional roles of the different sets of markers identified. The gene ontology categories identified for the transcripts of the ‘second order’ CpG sites of cg10151248-*PC* and cg13482620-*B3GNTL1*, showed a large degree of overlap for categories linked to immune responses. The genes and consequently the categories indicated for cg10151248-*PC* clearly differed from those derived from LC-AwS CpG sites (categories linked to response to external stressors). Results from cg13482620-*B3GNTL1* showed similarity with those from cg10151248-*PC* but also exhibited some common categories with LC-AwS sites. Thus indicating that a wide search provided novel information on potential pathways of relevance for lung cancer.

There is very limited evidence in the literature linking the methylation or expression levels at the two LC-non-AwS CpG sites and health outcomes. Notably, the CpG methylations of *PC* and *B3GNTL1* (located in unknown gene region and shelf region, respectively) were not associated with transcript expression for same genes. Nevertheless, hypermethylation at another CpG site in the gene *B3GNTL1* has been observed in colorectal tumors compared to adjacent tissue^39^ and the upregulated expression of this gene has been indicated as a potential marker for colorectal cancer^40^. Conversely, to the best of our knowledge there are no reported characterized description of the downstream consequences of altered methylation levels at cg10151248-*PC*.

Residual confounding by smoking in our adjusted analyses cannot be disregarded. However, CSI appeared to be a stringent adjustment for exposure to smoking and the argumentation above supports the manner in which we classified markers as being LC-AwS or LC-non-AwS (or not directly for cg13482620-*B3GNTL1*). Further, adjustment for estimates of white blood cell composition were not emphasized here due to the potential over-adjustment by smoking.

In conclusion, using blood-derived DNA methylation and gene expression profile from a prospective lung cancer study in Norwegian women, our study identified 25 differentially methylated CpG sites prior to lung cancer diagnosis, of which two appeared to be LC-non-AwS, in particular cg10151248-*PC*. These LC-non-AwS CpG sites seemed to be involved in biological pathways distinct from those related to LC-AwS CpG sites, and linked to immunological changes in blood prior to cancer diagnosis. Although the study size is limited, the use of a stringent significance level when assessing DNA methylation and gene expression data has revealed markers that represent prospective population-specific markers of smoking exposure as well as markers potentially relevant to lung cancer development and warrant further study.

## Electronic supplementary material

Supplementary Material

## Data Availability

The microarray data generated and/or analysed in the current study could be accessed upon reasonable request to the originating cohort. Access will be conditional to adherence to local ethical and security policy. R codes used for the analyses presented in the paper are available upon request.

## References

[1] Brothers JF (2013). Bridging the clinical gaps: genetic, epigenetic and transcriptomic biomarkers for the early detection of lung cancer in the post-National Lung Screening Trial era. BMC Med..

[2] Belinsky SA (2006). Promoter hypermethylation of multiple genes in sputum precedes lung cancer incidence in a high-risk cohort. Cancer Res..

[3] Lee SM, Park JY, Kim DS (2012). Methylation of TMEFF2 gene in tissue and serum DNA from patients with non-small cell lung cancer. Mol. Cells.

[4] Ulivi P (2006). p16(INK4A) and CDH13 hypermethylation in tumor and serum of non-small cell lung cancer patients. J. Cell. Physiol..

[5] Wang L (2010). Methylation markers for small cell lung cancer in peripheral blood leukocyte DNA. J. Thorac. Oncol..

[6] Fasanelli F (2015). Hypomethylation of smoking-related genes is associated with future lung cancer in four prospective cohorts. Nat. Commun..

[7] Baglietto L (2017). DNA methylation changes measured in pre-diagnostic peripheral blood samples are associated with smoking and lung cancer risk. Int. J. Cancer.

[8] Zhang Y (2016). Comparison and combination of blood DNA methylation at smoking-associated genes and at lung cancer-related genes in prediction of lung cancer mortality. Int. J. Cancer.

[9] Wang B-H (2017). Gene methylation as a powerful biomarker for detection and screening of non-small cell lung cancer in blood. Oncotarget.

[10] Shenker NS (2012). Epigenome-wide association study in the European Prospective Investigation into Cancer and Nutrition (EPIC-Turin) identifies novel genetic loci associated with smoking. Hum. Mol. Genet..

[11] Breitling LP, Yang R, Korn B, Burwinkel B, Brenner H (2011). Tobacco-smoking-related differential DNA methylation: 27K discovery and replication. Am. J. Hum. Genet..

[12] Besingi W, Johansson Å (2013). Smoke related DNA methylation changes in the etiology of human disease. Hum. Mol. Genet..

[13] Gao X, Jia M, Zhang Y, Breitling LP, Brenner H (2015). DNA methylation changes of whole blood cells in response to active smoking exposure in adults: a systematic review of DNA methylation studies. Clin. Epigenetics.

[14] Joehanes R (2016). Epigenetic signatures of cigarette smoking. Circ. Cardiovasc. Genet..

[15] Battram, T. *et al*. Appraising the causal relevance of DNA methylation for risk of lung cancer. *bioRxiv* **287888** (2018). 10.1093/ije/dyz190 PMC6857764 31549173

[16] Pallis AG, Syrigos KN (2013). Lung cancer in never smokers: disease characteristics and risk factors. Crit. Rev. Oncol. Hematol..

[17] Hu Y, Chen G (2015). Pathogenic mechanisms of lung adenocarcinoma in smokers and non-smokers determined by gene expression interrogation. Oncol. Lett..

[18] Zhang X, Gao L, Liu ZP, Jia S, Chen L (2016). Uncovering driver DNA methylation events in nonsmoking early stage lung adenocarcinoma. Biomed. Res. Int..

[19] Dumeaux V (2008). Gene expression analyses in breast cancer epidemiology: the Norwegian Women and Cancer postgenome cohort study. Breast Cancer Res..

[20] Dumeaux V (2010). Deciphering normal blood gene expression variation–The NOWAC postgenome study. PLoS Genet..

[21] Lund E (2008). Cohort profile: The Norwegian Women and Cancer Study–NOWAC–Kvinner og kreft. Int. J. Epidemiol..

[22] Baselmans BM (2015). Epigenome-wide association study of wellbeing. Twin Res. Hum. Genet..

[23] Willemsen G (2010). The Netherlands Twin Register biobank: a resource for genetic epidemiological studies. Twin Res. Hum. Genet..

[24] Guida F (2015). Dynamics of smoking-induced genome-wide methylation changes with time since smoking cessation. Hum. Mol. Genet..

[25] van Dongen J (2016). Genetic and environmental influences interact with age and sex in shaping the human methylome. Nat. Commun..

[26] Price EM (2013). Additional annotation enhances potential for biologically-relevant analysis of the Illumina Infinium HumanMethylation450 BeadChip array. Epigenetics Chromatin.

[27] Dumeaux V (2015). Peripheral blood cells inform on the presence of breast cancer: a population-based case-control study. Int. J. Cancer.

[28] Günther, C. C. *et al*. Preprocessing of gene-expression data related to breast cancer diagnosis. Report SAMBA/35/14Norwegian Computing Central available from: http://publications.nr.no/directdownload/directdownload/1415353311/preprocessinggunther.pdf. Accessed April 17, 2016 (2014).

[29] Chadeau-Hyam M (2014). Dynamics of the risk of smoking-induced lung cancer: a compartmental hidden Markov model for longitudinal analysis. Epidemiology.

[30] Houseman EA (2012). DNA methylation arrays as surrogate measures of cell mixture distribution. BMC Bioinformatics.

[31] Koestler DC (2013). Blood-based profiles of DNA methylation predict the underlying distribution of cell types: a validation analysis. Epigenetics.

[32] Leffondre K, Abrahamowicz M, Siemiatycki J, Rachet B (2002). Modeling smoking history: a comparison of different approaches. Am. J. Epidemiol..

[33] Forman, D. *et al*. Cancer incidence in five continents, Volume X. Lyon, Geneva: International Agency for Research on Cancer; Report distributed by World Health Organization Press available from: https://www.iarc.fr/en/publications/pdfs-online/epi/sp164/CI5volX_Full.pdf. 2014. Accessed October 22, 2016 (2014).

[34] Yu G, Wang L-G, Han Y, He Q-Y (2012). clusterProfiler: an R package for comparing biological themes among gene clusters. OMICS.

[35] Lee PN, Forey BA, Coombs KJ (2012). Systematic review with meta-analysis of the epidemiological evidence in the 1900s relating smoking to lung cancer. BMC Cancer.

[36] Li T (2015). Screening of lung cancer related SNPs and CNVs with SNP microarrays. Eur. Rev. Med. Pharmacol. Sci..

[37] Schübeler D (2015). Function and information content of DNA methylation. Nature.

[38] Huan T (2016). A whole-blood transcriptome meta-analysis identifies gene expression signatures of cigarette smoking. Hum. Mol. Genet..

[39] Berman BP (2012). Regions of focal DNA hypermethylation and long-range hypomethylation in colorectal cancer coincide with nuclear lamina-associated domains. Nat. Genet..

[40] Nagaraj SH, Reverter A (2011). A Boolean-based systems biology approach to predict novel genes associated with cancer: Application to colorectal cancer. BMC Syst. Biol..

