# Supplementary material for: DNA methylation and associated gene expression in blood prior to lung cancer diagnosis in the Norwegian Women and Cancer cohort
Source: Sci Rep. 2018 Nov 13;8:16714. doi: 10.1038/s41598-018-34334-6 (PMC6233189; doi:10.1038/s41598-018-34334-6)
Supplement: Supplementary file 1 — Supplementary Material [file 41598_2018_34334_MOESM1_ESM.pdf]

Supplementary Information for

DNA methylation and associated gene expression in blood prior to lung cancer diagnosis in the Norwegian Women and Cancer cohort

Torkjel Manning Sandanger[1, *, †], Therese Haugdahl Nøst[1, †], Florence Guida[2,3, †], Charlotta Rylander[1], Gianluca Campanella[2], David C. Muller[2], Jenny van Dongen[4], Dorret I. Boomsma[4], Mattias. Johansson[3], Paolo Vineis[2,5], Roel Vermeulen[2,6, ††], Eiliv Lund[1, ††], Marc Chadeau-Hyam[2,6, ††]

[1] Department of Community Medicine, Faculty of Health Sciences, UiT – The Arctic University of Norway, Tromsø, Norway.
[2] MRC/PHE Centre for Environmental Health, Department of Epidemiology and Biostatistics, Imperial College London, London, United Kingdom.
[3] Genetic Epidemiology Group, International Agency for Research on Cancer, Lyon, France
[4] Netherlands Twin Register, Vrije Universiteit, Department of Biological Psychology, Amsterdam, The Netherlands.
[5] Italian Institute for Genomic Medicine (IIGM), Turin, Italy.
[6] Institute for Risk Assessment Sciences (IRAS), Division of Environmental Epidemiology, Utrecht University, Utrecht, The Netherlands.

Table S1: Quantitative smoking metrics in lung cancer cases and age matched controls.

		Case N=131			Control N=129		
		Current (N=81)	Former (N=36)	Never (N=14)	Current (N=37)	Former (N=35)	Never (N=57)
**Duration (years)**	Mean	38.66	27.60	-	37.47	22.81	-
	St.dev	5.82	13.51	-	6.57	11.38	-
	Median	40.00	32.50	-	38.50	23.00	-
	Min	16.50	2.50	-	23.00	2.50	-
	Max	47.50	45.00	-	47.50	45.00	-
	NAs	1	1	-	3	1	-
**Cumulative mean intensity (cigarettes/day)**	Mean	12.60	12.05	-	11.70	8.18	-
	St.dev	4.55	5.68	-	4.03	3.77	-
	Median	12.00	12.00	-	11.36	7.00	-
	Min	2.50	2.50	-	4.00	2.50	-
	Max	27.00	24.05	-	20.94	17.00	-
	NAs	1	1	-	3	1	-
**Time since smoking cessation (years)**	Mean	-	10.28	-	-	14.05	-
	St.dev	-	11.16	-	-	11.34	-
	Median	-	5.00	-	-	12.00	-
	Min	-	0.50	-	-	1.00	-
	Max	-	40.00	-	-	39.50	-
	NAs	-	2	-	-	2	-
**CSI**	Mean	1.60	1.12	0	1.53	0.78	0
	St.dev	0.30	0.66	0	0.31	0.50	0
	Median	1.65	1.29	0	1.54	0.78	0
	Min	0.67	0.13	0	0.78	0.05	0
	Max	2.21	2.09	0	2.04	1.86	0
	NAs	1	2	-	3	2	-

CSI: Comprehensive Smoking Index (unitless)

Table S2: Summary description of estimated proportions of white blood cells in samples obtained from the Houseman algorithm.

Variable		Cases N=131	Controls N=129	Cases Current (N=81)	Cases Former (N=36)	Cases Never (N=14)	Controls Current (N=37)	Controls Former (N=35)	Controls Never (N=57)	Ca/Co[a] p-value	Smoking[b] p-value
Natural killer cells granulocytes	Mean	0.05	0.06	0.04	0.07	0.06	0.04	0.06	0.07	0.03	7.74E-07
	St.dev	0.04	0.06	0.04	0.04	0.04	0.05	0.05	0.06		
	Min	0.00	0.00	0	0	0	0	0	0		
	Max	0.21	0.24	0.21	0.17	0.13	0.24	0.21	0.23		
Lymphocytes B	Mean	0.06	0.06	0.06	0.05	0.06	0.06	0.06	0.06	0.65	0.53
	St.dev	0.03	0.02	0.03	0.02	0.03	0.02	0.02	0.02		
	Min	0.01	0.01	0.01	0.02	0.01	0.01	0.03	0.01		
	Max	0.23	0.12	0.23	0.11	0.1	0.1	0.1	0.12		
Lymphocytes T CD4	Mean	0.10	0.12	0.1	0.1	0.11	0.14	0.1	0.12	0.11	0.82
	St.dev	0.09	0.10	0.09	0.09	0.1	0.1	0.09	0.1		
	Min	0.00	0.00	0	0	0	0	0	0		
	Max	0.38	0.40	0.38	0.35	0.27	0.37	0.29	0.4		
Lymphocytes T CD8	Mean	0.22	0.21	0.23	0.21	0.2	0.2	0.23	0.21	0.51	0.82
	St.dev	0.11	0.12	0.11	0.11	0.11	0.09	0.12	0.12		
	Min	0.00	0.00	0	0.04	0	0	0	0		
	Max	0.57	0.50	0.57	0.49	0.35	0.39	0.47	0.5		
Monocytes granulocytes	Mean	0.06	0.06	0.06	0.06	0.06	0.07	0.07	0.06	0.56	0.63
	St.dev	0.03	0.03	0.03	0.03	0.03	0.03	0.03	0.02		
	Min	0.00	0.00	0	0	0	0.01	0.02	0		
	Max	0.13	0.14	0.13	0.13	0.13	0.12	0.14	0.14		
Eosinophils granulocytes	Mean	0.00	0.00	0	0	0	0	0	0	0.25	0.61
	St.dev	0.00	0.01	0	0	0	0.01	0	0		
	Min	0.00	0.00	0	0	0	0	0	0		
	Max	0.00	0.08	0	0	0	0.08	0	0.01		
Neutrophils granulocytes	Mean	0.53	0.51	0.54	0.52	0.53	0.52	0.51	0.51	0.15	0.32
	St.dev	0.10	0.10	0.1	0.1	0.11	0.11	0.1	0.1		
	Min	0.26	0.22	0.3	0.26	0.41	0.24	0.36	0.22		
	Max	0.81	0.75	0.76	0.74	0.81	0.75	0.69	0.74		

[a] Student t-test comparing means between cases and controls. [b]Kruskal Wallis test across the smoking categories current, former, never.

Table S3: Mean beta value and standard deviation for the CpGs associated to lung cancer in the NOWAC study and that were either un-associated with smoking (LC-non-AwS), or associated with smoking (LC-AwS).

ProbeID	Gene name	Chromosome	Cases N=131		Controls N=129	
			Mean	Standard deviation	Mean	Standard deviation
LC-Non-AwS CpG sites						
cg10151248	*PC*	11	0.95	0.02	0.96	0.01
cg13482620	*B3GNTL1*	17	0.84	0.02	0.85	0.03
LC-AwS CpG sites						
cg05575921	*AHRR*	5	0.65	0.12	0.78	0.12
cg03636183	*F2RL3*	19	0.55	0.09	0.62	0.08
cg06126421	*NA*	6	0.65	0.09	0.73	0.09
cg21566642	*NA*	2	0.39	0.09	0.47	0.10
cg02152091	*NA*	8	0.72	0.03	0.74	0.03
cg03898802	*DOPEY2*	21	0.91	0.02	0.92	0.02
cg06500852	*NA*	2	0.88	0.02	0.89	0.02
cg20024310	*NA*	7	0.75	0.03	0.76	0.03
cg06368429	*KPNA7*	7	0.74	0.03	0.76	0.03
cg02451831	*KIAA0087*	7	0.71	0.04	0.74	0.04
cg13936208	*NA*	12	0.85	0.02	0.86	0.02
cg13525026	*MYO15A*	17	0.88	0.02	0.90	0.02
cg08928494	*CA5A*	16	0.78	0.05	0.81	0.03
cg25305703	*NA*	8	0.68	0.07	0.74	0.07
cg00395990	*PDZD3*	11	0.79	0.04	0.80	0.04
cg25324976	*CSHL1*	17	0.83	0.02	0.85	0.02
cg01940273	*NA*	2	0.54	0.07	0.59	0.07
cg11635401	*MYO9B*	19	0.92	0.02	0.93	0.02
cg22475974	*NA*	4	0.97	0.01	0.97	0.01
cg21838013	*CRTAM*	11	0.73	0.04	0.74	0.03
cg23069177	*OCA2*	15	0.83	0.03	0.85	0.03
cg21161138	*AHRR*	5	0.66	0.07	0.71	0.06
cg16976547	*FES*	15	0.96	0.02	0.96	0.01

Table S4: The p-values for the covariate estimates in unconditional logistic regressions for the 25 candidate CpGs that were either un-associated with smoking (LC-non-AwS), or associated with smoking (LC-AwS). The p-values for the CpGs themselves are presented in Table 2 and S3.

**ProbeID**	**Gene name**	**Chromo-some**	**Smoking status - former** p-value	**Smoking status - current** p-value	**Packyears** p-value	**CSI** p-value	**Monocytes** p-value	**Lymphocyte B** p-value	**Neutrophils** p-value	**Lymphocyte CD4T** p-value	**Lymphocyte CD8T** p-value
LC-Non-AwS CpG sites											
cg10151248	*PC*	11	5.3E-04	**1.2E-08**	**3.9E-10**	1.1E-03	1.9E-01	5.1E-01	8.1E-01	5.8E-01	7.0E-01
cg13482620	*B3GNTL1*	17	2.5E-03	**5.5E-09**	**1.1E-10**	7.8E-06	4.5E-01	4.9E-01	3.1E-01	6.2E-01	7.5E-01
LC-AwS CpG sites											
cg05575921	*AHRR*	5	2.6E-02	4.6E-01	3.1E-03	**7.0E-09**	8.6E-01	1.0E-01	8.3E-02	9.4E-01	8.9E-01
cg03636183	*F2RL3*	19	1.1E-02	4.2E-02	5.8E-04	5.4E-05	2.6E-01	5.0E-01	6.1E-01	5.3E-01	8.1E-01
cg06126421	*NA*	6	1.4E-02	1.0E-02	2.3E-04	**1.2E-09**	8.3E-01	1.1E-01	7.9E-02	7.0E-01	4.5E-01
cg21566642	*NA*	2	8.0E-03	1.9E-02	1.8E-04	7.6E-07	3.7E-01	2.9E-01	8.7E-01	7.1E-01	6.4E-01
cg02152091	*NA*	8	7.7E-04	**1.5E-08**	**1.1E-09**	6.9E-03	2.5E-01	5.5E-01	9.5E-01	4.5E-01	7.2E-01
cg03898802	*DOPEY2*	21	9.6E-04	**6.6E-08**	**4.2E-09**	**1.9E-09**	2.6E-01	2.6E-01	9.0E-02	9.0E-01	8.0E-01
cg06500852	*NA*	2	1.0E-03	**1.1E-07**	**1.9E-08**	2.8E-02	1.2E-01	8.8E-01	7.2E-01	2.3E-01	3.2E-01
cg20024310	*NA*	7	1.9E-04	**9.5E-09**	**1.4E-09**	**2.0E-09**	4.2E-01	1.9E-01	8.7E-02	8.3E-01	6.8E-01
cg06368429	*KPNA7*	7	1.1E-03	**1.1E-07**	**1.6E-09**	**1.0E-08**	2.1E-01	1.8E-01	2.8E-01	5.2E-01	6.1E-01
cg02451831	*KIAA0087*	7	3.1E-04	3.3E-05	6.1E-07	**7.3E-10**	8.0E-01	1.3E-01	1.9E-02	4.9E-01	3.5E-01
cg13936208	*NA*	12	2.1E-03	**3.2E-08**	**9.9E-09**	**4.1E-10**	4.2E-01	6.4E-01	3.3E-01	6.7E-01	8.2E-01
cg13525026	*MYO15A*	17	8.0E-04	2.2E-07	**1.2E-08**	**1.5E-10**	3.9E-01	2.0E-01	3.3E-01	7.0E-01	9.3E-01
cg08928494	*CA5A*	16	5.6E-04	**3.5E-08**	**1.2E-09**	**3.3E-11**	2.2E-01	4.4E-01	2.5E-01	7.1E-01	9.3E-01
cg25305703	*NA*	8	1.0E-03	2.5E-05	5.6E-07	**9.6E-09**	8.8E-02	7.2E-01	4.0E-01	7.0E-01	9.1E-01
cg00395990	*PDZD3*	11	2.4E-03	1.2E-07	**1.3E-08**	**2.4E-09**	3.8E-01	3.9E-01	5.6E-01	5.7E-01	8.5E-01
cg25324976	*CSHL1*	17	7.0E-04	**5.1E-08**	**4.1E-09**	**1.6E-09**	4.2E-01	2.2E-01	9.5E-02	9.7E-01	9.3E-01
cg01940273	*NA*	2	8.6E-04	1.4E-03	9.9E-06	**5.0E-10**	8.7E-01	5.1E-02	1.1E-01	9.3E-01	6.5E-01
cg11635401	*MYO9B*	19	7.4E-04	**5.5E-09**	**3.9E-10**	3.8E-03	2.0E-01	5.8E-01	7.8E-01	2.4E-01	5.5E-01
cg22475974	*NA*	4	7.4E-04	**3.4E-08**	**2.6E-09**	**9.6E-10**	2.0E-01	2.1E-01	2.4E-01	8.7E-01	8.5E-01
cg21838013	*CRTAM*	11	5.4E-04	**2.9E-08**	**1.3E-09**	**1.7E-09**	6.5E-01	2.3E-01	1.8E-01	1.0E+00	7.4E-01
cg23069177	*OCA2*	15	5.9E-04	**1.9E-08**	**1.3E-09**	**7.0E-10**	7.0E-01	3.1E-01	4.5E-01	5.7E-01	7.6E-01
cg21161138	*AHRR*	5	9.8E-04	3.1E-04	4.0E-06	1.1E-06	4.2E-01	6.0E-01	3.0E-01	5.7E-01	5.5E-01
cg16976547	*FES*	15	2.7E-04	**3.8E-08**	**3.3E-09**	**2.4E-09**	3.2E-01	3.8E-01	2.5E-01	9.9E-01	8.2E-01

CSI: Comprehensive smoking index. The p-values presented in this table are estimated from unconditional logistic regressions including denoised DNA methylation levels (DNA methylation adjusted for technical covariates and matching variables; see Method section) as an independent variable and adjusted for selected covariates. Results are presented for separate models adjusted for smoking status, packyears, CSI and WBCs (monocytes, neutrophils, lymphocytes B, CD4T, CD8T in one model), respectively. Bolded numbers for p-values are considered significant using a Bonferroni threshold.

Table S5: Adjusted models for 25 Bonferroni significant CpG sites differentially methylated in cases as compared to controls (N=131 cases, 129 controls) that were either un-associated with smoking (LC-non-AwS), or associated with smoking (LC-AwS). Extends from Table 2 in the main text.

Probe ID	Gene name	Chromo-some	Adjusted model - WBCs			Adjusted model - CSI + WBCs		
			OR	95% CI	p-value	OR	95% CI	p-value
LC-Non-AwS CpG sites								
cg10151248	*PC*	11	0.36	0.25 - 0.51	**2.3E-08**	0.33	0.22 - 0.5	**8.4E-08**
cg13482620	*B3GNTL1*	17	0.41	0.3 - 0.57	1.4E-07	0.32	0.21 - 0.49	**8.3E-08**
LC-AwS CpG sites								
cg05575921	*AHRR*	5	0.36	0.27 - 0.49	**1.4E-10**	0.61	0.37 - 1	5.2E-02
cg03636183	*F2RL3*	19	0.39	0.28 - 0.53	**1.5E-09**	0.63	0.4 - 0.99	4.5E-02
cg06126421	*NA*	6	0.37	0.27 - 0.52	**2.6E-09**	0.68	0.43 - 1.08	1.0E-01
cg21566642	*NA*	2	0.42	0.31 - 0.56	**6.8E-09**	0.74	0.45 - 1.2	2.2E-01
cg02152091	*NA*	8	0.39	0.28 - 0.53	**6.9E-09**	0.43	0.31 - 0.61	1.6E-06
cg03898802	*DOPEY2*	21	0.38	0.27 - 0.52	**8.2E-09**	0.40	0.28 - 0.58	6.9E-07
cg06500852	*NA*	2	0.39	0.28 - 0.53	**8.7E-09**	0.44	0.31 - 0.62	4.0E-06
cg20024310	*NA*	7	0.36	0.25 - 0.52	**2.4E-08**	0.36	0.24 - 0.54	9.8E-07
cg06368429	*KPNA7*	7	0.37	0.26 - 0.53	**3.0E-08**	0.43	0.3 - 0.62	5.8E-06
cg02451831	*KIAA0087*	7	0.41	0.29 - 0.57	1.2E-07	0.56	0.39 - 0.81	1.8E-03
cg13936208	*NA*	12	0.39	0.28 - 0.55	**6.7E-08**	0.42	0.29 - 0.61	5.6E-06
cg13525026	*MYO15A*	17	0.42	0.31 - 0.57	**4.7E-08**	0.46	0.33 - 0.65	9.1E-06
cg08928494	*CA5A*	16	0.37	0.26 - 0.52	**2.4E-08**	0.39	0.27 - 0.57	9.8E-07
cg25305703	*NA*	8	0.44	0.32 - 0.6	2.2E-07	0.65	0.46 - 0.92	1.7E-02
cg00395990	*PDZD3*	11	0.39	0.28 - 0.54	**2.7E-08**	0.47	0.33 - 0.68	4.0E-05
cg25324976	*CSHL1*	17	0.43	0.32 - 0.6	2.1E-07	0.48	0.34 - 0.69	4.7E-05
cg01940273	*NA*	2	0.47	0.35 - 0.63	3.1E-07	0.86	0.57 - 1.31	4.9E-01
cg11635401	*MYO9B*	19	0.43	0.32 - 0.59	2.1E-07	0.41	0.28 - 0.58	8.4E-07
cg22475974	*NA*	4	0.45	0.34 - 0.61	3.0E-07	0.52	0.38 - 0.71	4.8E-05
cg21838013	*CRTAM*	11	0.41	0.3 - 0.58	1.7E-07	0.44	0.31 - 0.63	6.1E-06
cg23069177	*OCA2*	15	0.43	0.31 - 0.59	3.0E-07	0.42	0.29 - 0.6	2.8E-06
cg21161138	*AHRR*	5	0.45	0.33 - 0.61	2.6E-07	0.81	0.55 - 1.18	2.7E-01
cg16976547	*FES*	15	0.40	0.29 - 0.56	1.2E-07	0.45	0.31 - 0.64	1.5E-05

OR, odds ratio; CI, confidence interval. Regression models include denoised DNA methylation levels (DNA methylation adjusted for technical covariates and matching variables; see Method section) as an independent variable. Model results are presented for unconditional logistic regressions adjusted for white blood cell estimates (WBC; monocytes, neutrophils, lymphocytes B, CD4T, CD8T) and CSI + WBCs. Bolded p-values are significant according to the Bonferroni threshold.

Table S6: Stratified analyses for the 25 lung cancer related CpGs that were un-associated with smoking (LC-non-AwS), or associated with smoking (LC-AwS) for never smokers only (14 cases, 57 controls).

ProbeID	Gene name	Chromosome	Never smokers *N*=71		
			OR	95% CI	p-value
LC-Non-AwS CpG sites					
cg10151248	*PC*	11	0.36	0.17 - 0.77	**0.01**
cg13482620	*B3GNTL1*	17	0.31	0.14 - 0.67	**0.00**
LC-AwS CpG sites					
cg05575921	*AHRR*	5	0.27	0.03 - 2.14	0.21
cg03636183	*F2RL3*	19	1.22	0.32 - 4.68	0.77
cg06126421	*NA*	6	0.28	0.08 - 0.94	**0.04**
cg21566642	*NA*	2	0.50	0.18 - 1.4	0.19
cg02152091	*NA*	8	0.38	0.19 - 0.77	**0.01**
cg03898802	*DOPEY2*	21	0.33	0.16 - 0.68	**0.00**
cg06500852	*NA*	2	0.61	0.32 - 1.15	0.13
cg20024310	*NA*	7	0.25	0.1 - 0.63	**0.00**
cg06368429	*KPNA7*	7	0.50	0.23 - 1.06	0.07
cg02451831	*KIAA0087*	7	0.28	0.12 - 0.69	**0.01**
cg13936208	*NA*	12	0.79	0.43 - 1.45	0.45
cg13525026	*MYO15A*	17	0.46	0.24 - 0.89	**0.02**
cg08928494	*CA5A*	16	0.36	0.16 - 0.79	**0.01**
cg25305703	*NA*	8	0.53	0.26 - 1.08	0.08
cg00395990	*PDZD3*	11	0.56	0.28 - 1.1	0.09
cg25324976	*CSHL1*	17	0.42	0.19 - 0.91	**0.03**
cg01940273	*NA*	2	0.66	0.27 - 1.58	0.35
cg11635401	*MYO9B*	19	0.64	0.35 - 1.16	0.14
cg22475974	*NA*	4	0.49	0.25 - 0.98	**0.04**
cg21838013	*CRTAM*	11	0.95	0.47 - 1.9	0.88
cg23069177	*OCA2*	15	0.43	0.2 - 0.89	**0.02**
cg21161138	*AHRR*	5	1.08	0.38 - 3.1	0.88
cg16976547	*FES*	15	0.64	0.29 - 1.44	0.28

Table S7: Stratified analyses for the 25 lung cancer related CpGs that were un-associated with smoking (LC-non-AwS), or associated with smoking (LC-AwS) for the histological subtypes and time to diagnosis.

**ProbeID**	**Gene name**	**Chromo-some**	**Adenocarcinomas**		**Other subtypes**		**Squamous + small cell subtypes**		**Short time to diagnosis**		**Long time to diagnosis**	
			OR	p-value	OR	p-value	OR	p-value	OR	p-value	OR	p-value
LC-Non-AwS CpG sites												
cg10151248	*PC*	11	0.36	2.8E-06	0.36	2.1E-06	0.39	3.3E-05	0.33	1.4E-06	0.41	1.5E-05
cg13482620	*B3GNTL1*	17	0.35	4.5E-07	0.49	1.7E-04	0.54	4.6E-03	0.42	6.9E-06	0.42	2.5E-05
LC-AwS CpG sites												
cg05575921	*AHRR*	5	0.44	2.3E-06	0.31	**5.8E-10**	0.24	**4.1E-09**	0.35	**2.7E-09**	0.41	8.3E-07
cg03636183	*F2RL3*	19	0.49	2.5E-05	0.29	**6.4E-10**	0.23	**7.1E-09**	0.34	**4.4E-09**	0.44	6.5E-06
cg06126421	*NA*	6	0.43	4.4E-06	0.35	**3.0E-08**	0.30	1.9E-07	0.35	**2.8E-08**	0.43	6.3E-06
cg21566642	*NA*	2	0.51	7.7E-05	0.34	**2.4E-08**	0.26	1.9E-07	0.37	**8.3E-08**	0.48	4.3E-05
cg02152091	*NA*	8	0.36	2.6E-07	0.44	7.1E-06	0.45	1.2E-04	0.39	6.8E-07	0.41	3.6E-06
cg03898802	*DOPEY2*	21	0.34	4.8E-07	0.42	2.6E-06	0.36	3.6E-06	0.35	1.2E-07	0.42	1.3E-05
cg06500852	*NA*	2	0.38	1.2E-06	0.42	2.1E-06	0.38	4.6E-06	0.37	1.7E-06	0.44	5.7E-06
cg20024310	*NA*	7	0.32	6.3E-07	0.41	1.1E-05	0.42	1.9E-04	0.34	1.0E-06	0.39	1.5E-05
cg06368429	*KPNA7*	7	0.40	6.0E-06	0.36	1.2E-06	0.35	9.5E-06	0.36	3.0E-07	0.42	3.0E-05
cg02451831	*KIAA0087*	7	0.41	4.1E-06	0.42	1.9E-06	0.39	1.5E-05	0.41	2.2E-06	0.41	2.5E-06
cg13936208	*NA*	12	0.36	2.0E-06	0.40	5.8E-06	0.37	4.1E-05	0.30	1.6E-07	0.47	1.7E-04
cg13525026	*MYO15A*	17	0.48	4.3E-05	0.41	7.0E-07	0.39	4.9E-06	0.43	2.5E-06	0.46	2.4E-05
cg08928494	*CA5A*	16	0.33	7.9E-07	0.42	9.5E-06	0.36	2.6E-05	0.31	1.6E-07	0.43	6.9E-05
cg25305703	*NA*	8	0.49	4.3E-05	0.39	4.7E-07	0.37	4.2E-06	0.49	2.3E-05	0.40	1.2E-06
cg00395990	*PDZD3*	11	0.45	1.7E-05	0.39	1.9E-06	0.31	2.4E-06	0.47	1.7E-05	0.38	3.3E-06
cg25324976	*CSHL1*	17	0.46	2.2E-05	0.43	5.1E-06	0.36	6.0E-06	0.45	1.7E-05	0.43	9.6E-06
cg01940273	*NA*	2	0.56	5.5E-04	0.37	**1.0E-07**	0.33	1.8E-06	0.44	3.1E-06	0.47	3.4E-05
cg11635401	*MYO9B*	19	0.45	2.4E-05	0.43	6.5E-06	0.42	3.3E-05	0.40	1.4E-06	0.49	1.3E-04
cg22475974	*NA*	4	0.44	6.3E-06	0.47	2.1E-05	0.43	3.1E-05	0.46	1.9E-05	0.46	1.8E-05
cg21838013	*CRTAM*	11	0.42	1.2E-05	0.42	1.4E-05	0.38	4.8E-05	0.34	2.9E-07	0.51	6.6E-04
cg23069177	*OCA2*	15	0.44	1.5E-05	0.43	1.1E-05	0.47	2.7E-04	0.53	6.0E-04	0.35	4.1E-07
cg21161138	*AHRR*	5	0.49	3.3E-05	0.41	1.7E-06	0.36	4.1E-06	0.41	6.7E-07	0.50	1.6E-04
cg16976547	*FES*	15	0.38	7.9E-07	0.47	8.7E-05	0.43	1.7E-04	0.41	5.0E-06	0.45	2.4E-05

Bolded numbers for p-values are considered significant using a Bonferroni threshold. Time to diagnosis was stratified according to it's median of 4.2 years.

Table S8: List of the significant CpG-transcript pairs identified for the 'second order' CpGs that were associated to cg10151248-*PC*.

CpG ID	p-value	r	Transcript ID	Gene Name	Transcript	RefSeq ID
cg21570493	3.44E-13	-0.44	ILMN_37874	*CD79B*	ILMN_2366212	NM_001039933.1
cg21570493	3.47E-13	-0.44	ILMN_138839	*CD79B*	ILMN_1785439	NM_021602.1
cg21570493	1.53E-12	-0.43	ILMN_306934	*BANK1*	ILMN_1661646	NM_017935.2
cg21570493	1.71E-12	-0.43	ILMN_11112	*OSBPL10*	ILMN_1669497	NM_017784.3
cg21570493	1.20E-11	-0.42	ILMN_27934	*EBF1*	ILMN_1778681	NM_024007.2
cg21570493	1.66E-11	-0.41	ILMN_2004	*CD19*	ILMN_1782704	NM_001770.3
cg21570493	9.94E-11	-0.40	ILMN_22155	*TNFRSF13C*	ILMN_1731742	NM_052945.2
cg21570493	1.17E-10	-0.40	ILMN_139125	*CD79B*	ILMN_1710017	NM_000626.1
cg21570493	2.86E-10	-0.39	ILMN_25438	*MS4A1*	ILMN_2401714	NM_021950.3
cg21570493	4.31E-10	-0.39	ILMN_26827	*FAM129C*	ILMN_1664063	NM_173544.2
cg21570493	8.17E-10	-0.38	ILMN_17359	*BCL11A*	ILMN_1752899	NM_022893.2
cg21570493	8.68E-10	-0.38	ILMN_10080	*LOC90925*	ILMN_1794927	NM_175870.3
cg21570493	1.01E-09	-0.38	ILMN_37677	*CD79A*	ILMN_1734878	NM_001783.2
cg21570493	1.03E-09	-0.38	ILMN_19896	*CXCR5*	ILMN_2337928	NM_032966.1
cg21570493	1.44E-09	-0.37	ILMN_162315	*POU2AF1*	ILMN_1811049	NM_006235.1
cg21570493	3.21E-09	-0.37	ILMN_23414	*BLK*	ILMN_1668277	NM_001715.2
cg21570493	5.41E-09	-0.36	ILMN_22421	*CR2*	ILMN_2369666	NM_001877.3
cg21570493	1.30E-08	-0.35	ILMN_176442	*CCR6*	ILMN_1690907	NM_031409.2
cg21570493	1.50E-08	-0.35	ILMN_28506	*PARM1*	ILMN_1656560	NM_015393.2

Table S9: List of the unique CpG-transcript pairs identified for the 'second order' CpGs that were associated to cg13482620-*B3GNTL1*.

CpG ID	p-value	r	Transcript ID	Gene Name	Transcript	RefSeq ID
cg03160057	1.73E-14	0.46	ILMN_27115	*NDUFB8*	ILMN_1661170	NM_005004.2
cg03160057	1.82E-14	-0.46	ILMN_167573	*SIPA1L2*	ILMN_1732923	NM_020808.3
cg03160057	2.40E-14	-0.46	ILMN_164363	*KCNJ15*	ILMN_1675756	NM_170736.1
cg03160057	2.72E-14	-0.46	ILMN_7658	*CASP4*	ILMN_1678454	NM_001225.3
cg03160057	4.79E-14	-0.45	ILMN_5816	*RALB*	ILMN_1676358	NM_002881.2
cg10115918	8.26E-14	-0.45	ILMN_11112	*OSBPL10*	ILMN_1669497	NM_017784.3
cg03160057	2.86E-13	-0.44	ILMN_20362	*NDEL1*	ILMN_1705064	NM_030808.3
cg03160057	3.86E-13	-0.44	ILMN_167872	*SLC22A4*	ILMN_2050911	NM_003059.2
cg03160057	4.67E-13	0.44	ILMN_111893	*HS.554324*	ILMN_1886655	
cg03160057	1.01E-12	0.43	ILMN_23399	*TUBB*	ILMN_2101885	NM_178014.2
cg03160057	1.16E-12	-0.43	ILMN_28019	*PHF21A*	ILMN_1699496	NM_016621.2
cg03160057	1.90E-12	-0.43	ILMN_17922	*FCAR*	ILMN_2379967	NM_133271.1
cg03160057	2.24E-12	-0.43	ILMN_10853	*ABHD5*	ILMN_1655702	NM_016006.3
cg03160057	3.28E-12	0.42	ILMN_370218	*LOC552889*	ILMN_3237507	NM_001136262.1
cg03160057	3.43E-12	-0.42	ILMN_15164	*AQP9*	ILMN_1715068	NM_020980.2
cg03160057	3.65E-12	-0.42	ILMN_307349	*ENTPD1*	ILMN_1773125	NM_001098175.1
cg03160057	3.83E-12	-0.42	ILMN_26692	*MGAM*	ILMN_1714643	NM_004668.1
cg03160057	4.01E-12	-0.42	ILMN_163517	*LIMK2*	ILMN_1687960	NM_016733.2
cg03160057	4.02E-12	0.42	ILMN_25658	*AES*	ILMN_1712944	NM_001130.5
cg03160057	4.17E-12	-0.42	ILMN_12367	*ACSL1*	ILMN_1684585	NM_001995.2
cg03160057	4.72E-12	-0.42	ILMN_20343	*SLC26A8*	ILMN_1755843	NM_052961.2
cg03160057	5.62E-12	-0.42	ILMN_5773	*B4GALT5*	ILMN_1685824	NM_004776.2
cg03160057	5.99E-12	0.42	ILMN_1662	*PHB*	ILMN_1692651	NM_002634.2
cg03160057	7.09E-12	-0.42	ILMN_15638	*TLR6*	ILMN_1654560	NM_006068.2
cg03160057	7.18E-12	-0.42	ILMN_165233	*ANTXR2*	ILMN_1812926	NM_058172.3
cg03160057	8.22E-12	-0.42	ILMN_15812	*SLC2A3*	ILMN_1775708	NM_006931.1
cg03160057	8.35E-12	0.42	ILMN_8267	*PTPRCAP*	ILMN_1672417	NM_005608.2
cg03160057	9.45E-12	-0.42	ILMN_6510	*QPCT*	ILMN_1741727	NM_012413.3
cg03160057	1.11E-11	-0.41	ILMN_25291	*NPL*	ILMN_2149494	NM_030769.1
cg03160057	1.11E-11	0.41	ILMN_163755	*LFNG*	ILMN_1663080	NM_001040167.1
cg10115918	1.78E-11	-0.41	ILMN_139125	*CD79B*	ILMN_1710017	NM_000626.1
cg03160057	2.02E-11	-0.41	ILMN_15983	*NLRC4*	ILMN_1796976	NM_021209.3
cg03160057	2.20E-11	-0.41	ILMN_18651	*GPR97*	ILMN_1765941	NM_170776.3
cg10115918	2.31E-11	-0.41	ILMN_27934	*EBF1*	ILMN_1778681	NM_024007.2
cg03160057	2.39E-11	0.41	ILMN_32693	*C17ORF90*	ILMN_2043615	NM_001039842.1
cg05664421	2.54E-11	-0.41	ILMN_162734	*LOC730820*	ILMN_1719344	XM_001127763.1
cg03160057	2.61E-11	-0.41	ILMN_19052	*NT5C2*	ILMN_1682165	NM_012229.2
cg03160057	2.74E-11	-0.41	ILMN_10003	*GMPR2*	ILMN_2365595	NM_001002000.1
cg03160057	3.09E-11	-0.41	ILMN_29514	*ADM*	ILMN_1708934	NM_001124.1
cg03160057	3.14E-11	-0.41	ILMN_18397	*MCL1*	ILMN_1756806	NM_021960.3
cg13752749	3.25E-11	-0.41	ILMN_308315	*MIAT*	ILMN_1864900	NR_003491.1
cg03160057	3.44E-11	0.40	ILMN_19480	*TBC1D10C*	ILMN_1710434	NM_198517.2
cg03160057	3.70E-11	-0.40	ILMN_18651	*GPR97*	ILMN_1815054	NM_170776.3
cg03160057	3.97E-11	0.40	ILMN_25454	*ESYT1*	ILMN_1761159	NM_015292.1
cg03160057	4.04E-11	-0.40	ILMN_3601	*SLC22A4*	ILMN_1685057	NM_003059.2
cg01676996	4.07E-11	-0.40	ILMN_335816	*LOC645638*	ILMN_3200140	XR_040455.1
cg03160057	4.44E-11	-0.40	ILMN_26366	*FCGR2A*	ILMN_1706523	NM_021642.2
cg03160057	4.48E-11	-0.40	ILMN_1351	*MAPK1*	ILMN_1767320	NM_138957.2
cg11654904	4.67E-11	-0.40	ILMN_43946	*LOC644039*	ILMN_1661917	XM_929431.1
cg03160057	4.75E-11	0.40	ILMN_40941	*LOC649447*	ILMN_1811063	XM_941125.1
cg03160057	4.81E-11	0.40	ILMN_11685	*SNRPN*	ILMN_2372082	NM_022805.2
cg03160057	5.40E-11	-0.40	ILMN_21688	*MME*	ILMN_1678170	NM_000902.3
cg03160057	5.41E-11	-0.40	ILMN_14880	*NFIL3*	ILMN_1707312	NM_005384.2
cg03160057	5.43E-11	-0.40	ILMN_163833	*PFKFB3*	ILMN_2186061	NM_004566.2
cg05664421	6.80E-11	-0.40	ILMN_15638	*TLR6*	ILMN_1654560	NM_006068.2
cg03160057	7.01E-11	-0.40	ILMN_174069	*KIF1B*	ILMN_1735552	NM_015074.2

cg03160057	7.04E-11	0.40	ILMN_29102	*EVL*	ILMN_1730622	NM_016337.2
cg10115918	7.39E-11	-0.40	ILMN_162315	*POU2AF1*	ILMN_1811049	NM_006235.1
cg03160057	8.88E-11	-0.40	ILMN_29203	*TRIB1*	ILMN_1803811	NM_025195.2
cg03160057	9.72E-11	-0.40	ILMN_8722	*FCAR*	ILMN_2365091	NM_133280.1
cg03160057	9.74E-11	-0.40	ILMN_4100	*LMNB1*	ILMN_2126706	NM_005573.2
cg03160057	1.04E-10	-0.40	ILMN_180663	*NCOA1*	ILMN_1669033	NM_147223.2
cg03160057	1.34E-10	0.39	ILMN_27913	*POMGNT1*	ILMN_1693664	NM_017739.2
cg10115918	1.40E-10	-0.39	ILMN_23414	*BLK*	ILMN_1668277	NM_001715.2
cg03160057	1.40E-10	0.39	ILMN_26865	*NXT1*	ILMN_1760280	NM_013248.2
cg03160057	1.48E-10	-0.39	ILMN_26097	*IL10RB*	ILMN_1767360	NM_000628.3
cg05664421	1.57E-10	0.39	ILMN_169915	*WDR74*	ILMN_1809866	XM_001125771.1
cg03160057	1.62E-10	0.39	ILMN_6297	*C10ORF33*	ILMN_1684497	NM_032709.1
cg11654904	1.67E-10	-0.39	ILMN_20452	*LRBA*	ILMN_2153373	NM_006726.1
cg03160057	1.67E-10	0.39	ILMN_12457	*RASSF7*	ILMN_1733110	NM_003475.2
cg03160057	1.77E-10	-0.39	ILMN_33519	*LOC642103*	ILMN_1712999	XM_936233.1
cg03160057	1.77E-10	-0.39	ILMN_28962	*BASP1*	ILMN_1651826	NM_006317.3
cg03160057	1.83E-10	-0.39	ILMN_8124	*DIRC2*	ILMN_1793743	NM_032839.1
cg03160057	2.15E-10	0.39	ILMN_1993	*SAE1*	ILMN_1657204	NM_005500.1
cg03160057	2.33E-10	-0.39	ILMN_34755	*TBC1D24*	ILMN_1780197	NM_020705.1
cg03160057	2.53E-10	-0.39	ILMN_10394	*SH3GLB1*	ILMN_1766045	NM_016009.2
cg05664421	2.78E-10	-0.39	ILMN_26866	*TMEM49*	ILMN_1692754	NM_030938.2
cg10115918	2.84E-10	-0.39	ILMN_165620	*TNFRSF13B*	ILMN_1759075	NM_012452.2
cg10115918	2.85E-10	-0.39	ILMN_25438	*MS4A1*	ILMN_2401714	NM_021950.3
cg03160057	2.87E-10	-0.39	ILMN_540	*BCL6*	ILMN_1737314	NM_001706.2
cg03160057	2.95E-10	-0.39	ILMN_177429	*HAL*	ILMN_2074748	NM_002108.2
cg03160057	3.04E-10	0.39	ILMN_370567	*ZBTB42*	ILMN_3239445	NM_001137601.1
cg03160057	3.05E-10	-0.39	ILMN_22627	*PYGL*	ILMN_1696187	NM_002863.3
cg03160057	3.10E-10	0.39	ILMN_29912	*ZAP70*	ILMN_1719756	NM_001079.3
cg05664421	3.17E-10	0.39	ILMN_1662	*PHB*	ILMN_1692651	NM_002634.2
cg04909834	3.18E-10	-0.39	ILMN_11112	*OSBPL10*	ILMN_1669497	NM_017784.3
cg05664421	3.37E-10	0.39	ILMN_40941	*LOC649447*	ILMN_1811063	XM_941125.1
cg03160057	3.40E-10	0.39	ILMN_17720	*NHP2L1*	ILMN_1763460	NM_001003796.1
cg10115918	3.47E-10	-0.39	ILMN_37874	*CD79B*	ILMN_2366212	NM_001039933.1
cg03160057	3.62E-10	0.38	ILMN_19173	*FAM62B*	ILMN_2057573	NM_020728.1
cg10115918	3.72E-10	-0.38	ILMN_306934	*BANK1*	ILMN_1661646	NM_001083907.1
cg03160057	3.84E-10	-0.38	ILMN_167352	*RNF149*	ILMN_2112524	NM_173647.2
cg03160057	3.98E-10	0.38	ILMN_23763	*SKAP1*	ILMN_1751400	NM_003726.3
cg06836020	4.10E-10	0.38	ILMN_12782	*GIYD1*	ILMN_2368575	NM_001014999.1
cg03160057	4.19E-10	0.38	ILMN_1871	*CCDC130*	ILMN_1758633	NM_030818.2
cg03160057	4.24E-10	-0.38	ILMN_25336	*NUP50*	ILMN_1725612	NM_007172.3
cg03160057	4.25E-10	-0.38	ILMN_37027	*LOC654103*	ILMN_1802808	XM_939368.1
cg03160057	4.27E-10	-0.38	ILMN_14535	*SDCBP*	ILMN_2363586	NM_001007067.1
cg03160057	4.34E-10	0.38	ILMN_17661	*SPNS3*	ILMN_1668984	NM_182538.3
cg03160057	4.46E-10	-0.38	ILMN_32201	*LOC653778*	ILMN_1707434	XM_929667.1
cg03160057	4.86E-10	0.38	ILMN_27010	*GIMAP5*	ILMN_1769383	NM_018384.3
cg11654904	5.11E-10	-0.38	ILMN_169268	*CNOT1*	ILMN_1669206	NM_016284.3
cg03160057	5.24E-10	0.38	ILMN_15060	*SLC25A25*	ILMN_1791728	NM_052901.2
cg11654904	5.28E-10	-0.38	ILMN_23763	*SKAP1*	ILMN_1751400	NM_003726.3
cg03160057	5.35E-10	0.38	ILMN_306788	*COMMD7*	ILMN_1810334	NM_001099339.1
cg10115918	5.39E-10	-0.38	ILMN_138839	*CD79B*	ILMN_1785439	NM_021602.1
cg03160057	5.55E-10	0.38	ILMN_1174	*TRAPPC6A*	ILMN_1775703	NM_024108.1
cg03160057	5.68E-10	0.38	ILMN_29564	*AFG3L2*	ILMN_2066124	NM_006796.1
cg05664421	6.14E-10	-0.38	ILMN_10320	*RNF149*	ILMN_1665877	NM_173647.2
cg03160057	6.44E-10	0.38	ILMN_864	*TMEM203*	ILMN_2073010	NM_053045.1
cg03160057	6.48E-10	0.38	ILMN_11698	*QARS*	ILMN_1763080	NM_005051.1
cg03160057	6.68E-10	-0.38	ILMN_25291	*NPL*	ILMN_1782070	NM_030769.1
cg03160057	7.60E-10	-0.38	ILMN_21293	*ETS2*	ILMN_1720158	NM_005239.4
cg03160057	8.18E-10	0.38	ILMN_41835	*LOC643433*	ILMN_1661306	XM_928197.1
cg03160057	8.27E-10	-0.38	ILMN_23197	*STAU1*	ILMN_1656136	NM_004602.2
cg10115918	8.62E-10	-0.38	ILMN_12678	*COBLL1*	ILMN_1761260	NM_014900.3

cg03160057	8.63E-10	0.38	ILMN_28665	*BTBD6*	ILMN_1744725	NM_033271.1
cg03160057	8.88E-10	-0.38	ILMN_162321	*CREBBP*	ILMN_1809583	NM_001079846.1
cg03160057	9.29E-10	0.38	ILMN_29979	*TMED3*	ILMN_1719316	NM_007364.2
cg05664421	9.91E-10	0.38	ILMN_11009	*RLTPR*	ILMN_1746138	NM_001013838.1
cg03160057	9.97E-10	-0.38	ILMN_23908	*SLC2A14*	ILMN_1668865	NM_153449.2
cg03160057	1.02E-09	0.38	ILMN_3512	*STMN3*	ILMN_3244117	NM_015894.2
cg03160057	1.08E-09	-0.37	ILMN_162734	*LOC730820*	ILMN_1719344	XM_001127763.1
cg11654904	1.08E-09	0.37	ILMN_165748	*SORT1*	ILMN_1707077	NM_002959.4
cg03160057	1.11E-09	0.37	ILMN_176427	*TYSND1*	ILMN_1775677	NM_001040273.1
cg01676996	1.13E-09	-0.37	ILMN_95640	*HS.478682*	ILMN_1872404	
cg03160057	1.15E-09	-0.37	ILMN_27776	*FLJ22662*	ILMN_1707286	NM_024829.4
cg03160057	1.19E-09	-0.37	ILMN_40785	*CR1*	ILMN_2388112	NM_000573.3
cg03160057	1.20E-09	0.37	ILMN_164731	*SFI1*	ILMN_1763887	NM_001007467.1
cg27665823	1.23E-09	-0.37	ILMN_2166	*SDAD1*	ILMN_1735360	NM_018115.2
cg05664421	1.24E-09	-0.37	ILMN_18859	*STX3*	ILMN_1659544	NM_004177.3
cg11654904	1.26E-09	-0.37	ILMN_2166	*SDAD1*	ILMN_1735360	NM_018115.2
cg05664421	1.28E-09	0.37	ILMN_25454	*ESYT1*	ILMN_1761159	NM_015292.1
cg03160057	1.29E-09	-0.37	ILMN_7898	*LITAF*	ILMN_1713934	NM_004862.2
cg03160057	1.30E-09	0.37	ILMN_6032	*PAICS*	ILMN_2392546	NM_006452.3
cg03160057	1.32E-09	-0.37	ILMN_8741	*FCGR3B*	ILMN_2134453	NM_000570.2
cg03160057	1.37E-09	-0.37	ILMN_17196	*EPB41L5*	ILMN_1770245	NM_020909.2

Table S10: Gene ontology categories significant in overrepresentation analysis of transcripts related to cg10151248-*PC*.

ID	Description	GeneRatio	p-value
GO:0042113	B cell activation	8 / 15	9E-12
GO:0046649	lymphocyte activation	8 / 15	4E-08
GO:0045321	leukocyte activation	8 / 15	1E-07
GO:0050778	positive regulation of immune response	6 / 15	3E-05
GO:0002250	adaptive immune response	5 / 15	2E-05
GO:0002429	immune response-activating cell surface receptor signaling pathway	5 / 15	2E-05
GO:0002768	immune response-regulating cell surface receptor signaling pathway	5 / 15	3E-05
GO:0002757	immune response-activating signal transduction	5 / 15	9E-05
GO:0002253	activation of immune response	5 / 15	1E-04
GO:0002764	immune response-regulating signaling pathway	5 / 15	1E-04
GO:0050853	B cell receptor signaling pathway	4 / 15	3E-07
GO:0042100	B cell proliferation	4 / 15	9E-07
GO:0006959	humoral immune response	4 / 15	8E-06
GO:0032943	mononuclear cell proliferation	4 / 15	6E-05
GO:0046651	lymphocyte proliferation	4 / 15	6E-05
GO:0070661	leukocyte proliferation	4 / 15	8E-05
GO:0050851	antigen receptor-mediated signaling pathway	4 / 15	9E-05
GO:0030098	lymphocyte differentiation	4 / 15	2E-04
GO:0002521	leukocyte differentiation	4 / 15	1E-03
GO:0030183	B cell differentiation	3 / 15	1E-04
GO:0002460	adaptive immune response based on somatic recombination of immune receptors built from immunoglobulin superfamily domains	3 / 15	1E-03
GO:0019221	cytokine-mediated signaling pathway	3 / 15	1E-02
GO:0071345	cellular response to cytokine stimulus	3 / 15	3E-02

Table S11: Gene ontology categories significant in overrepresentation analysis of transcripts related to cg13482620-*B3GNTL1*.

ID	Description	GeneRatio	p-value
GO:0002253	activation of immune response	11 / 107	0.002
GO:0050778	positive regulation of immune response	11 / 107	0.007
GO:0045321	leukocyte activation	10 / 107	0.033
GO:0042113	B cell activation	9 / 107	0.000
GO:1902532	negative regulation of intracellular signal transduction	9 / 107	0.005
GO:0002757	immune response-activating signal transduction	9 / 107	0.009
GO:0002764	immune response-regulating signaling pathway	9 / 107	0.015
GO:0010638	positive regulation of organelle organization	9 / 107	0.018
GO:0046649	lymphocyte activation	9 / 107	0.031
GO:0010876	lipid localization	8 / 107	0.001
GO:0002429	immune response-activating cell surface receptor signaling pathway	8 / 107	0.004
GO:0002768	immune response-regulating cell surface receptor signaling pathway	8 / 107	0.008
GO:0044089	positive regulation of cellular component biogenesis	8 / 107	0.013
GO:0019693	ribose phosphate metabolic process	8 / 107	0.029
GO:0006869	lipid transport	7 / 107	0.003
GO:0002250	adaptive immune response	7 / 107	0.012
GO:0009991	response to extracellular stimulus	7 / 107	0.018
GO:0016485	protein processing	6 / 107	0.005
GO:0051604	protein maturation	6 / 107	0.010
GO:0009126	purine nucleoside monophosphate metabolic process	6 / 107	0.018
GO:0009167	purine ribonucleoside monophosphate metabolic process	6 / 107	0.018
GO:0009161	ribonucleoside monophosphate metabolic process	6 / 107	0.021
GO:0009123	nucleoside monophosphate metabolic process	6 / 107	0.025
GO:0017038	protein import	6 / 107	0.028
GO:0031667	response to nutrient levels	6 / 107	0.038
GO:0002683	negative regulation of immune system process	6 / 107	0.046
GO:0030183	B cell differentiation	5 / 107	0.001
GO:0050864	regulation of B cell activation	5 / 107	0.001
GO:0006959	humoral immune response	5 / 107	0.003
GO:1902115	regulation of organelle assembly	5 / 107	0.004
GO:0008643	carbohydrate transport	5 / 107	0.010
GO:0050851	antigen receptor-mediated signaling pathway	5 / 107	0.033
GO:0015711	organic anion transport	5 / 107	0.039
GO:0006606	protein import into nucleus	5 / 107	0.048
GO:0044744	protein targeting to nucleus	5 / 107	0.048
GO:1902593	single-organism nuclear import	5 / 107	0.048

GO:0050869	negative regulation of B cell activation	4 / 107	0.000
GO:0050853	B cell receptor signaling pathway	4 / 107	0.001
GO:1902117	positive regulation of organelle assembly	4 / 107	0.001
GO:0051250	negative regulation of lymphocyte activation	4 / 107	0.008
GO:0002695	negative regulation of leukocyte activation	4 / 107	0.014
GO:0009952	anterior/posterior pattern specification	4 / 107	0.021
GO:0050866	negative regulation of cell activation	4 / 107	0.021
GO:0015758	glucose transport	4 / 107	0.022
GO:0008645	hexose transport	4 / 107	0.023
GO:0015749	monosaccharide transport	4 / 107	0.023
GO:0071219	cellular response to molecule of bacterial origin	4 / 107	0.030
GO:0043393	regulation of protein binding	4 / 107	0.044
GO:0071216	cellular response to biotic stimulus	4 / 107	0.048
GO:0031668	cellular response to extracellular stimulus	4 / 107	0.049
GO:0009948	anterior/posterior axis specification	3 / 107	0.001
GO:0060291	long-term synaptic potentiation	3 / 107	0.002
GO:0009112	nucleobase metabolic process	3 / 107	0.002
GO:0060711	labyrinthine layer development	3 / 107	0.003
GO:0009798	axis specification	3 / 107	0.012
GO:0035282	segmentation	3 / 107	0.014
GO:0070613	regulation of protein processing	3 / 107	0.016
GO:1903317	regulation of protein maturation	3 / 107	0.016
GO:0001892	embryonic placenta development	3 / 107	0.016
GO:0042100	B cell proliferation	3 / 107	0.018
GO:0006641	triglyceride metabolic process	3 / 107	0.020
GO:0032091	negative regulation of protein binding	3 / 107	0.021
GO:0032092	positive regulation of protein binding	3 / 107	0.021
GO:0042273	ribosomal large subunit biogenesis	3 / 107	0.021
GO:0050806	positive regulation of synaptic transmission	3 / 107	0.024
GO:0016575	histone deacetylation	3 / 107	0.026
GO:1900034	regulation of cellular response to heat	3 / 107	0.030
GO:0006639	acylglycerol metabolic process	3 / 107	0.031
GO:0006638	neutral lipid metabolic process	3 / 107	0.032
GO:0006476	protein deacetylation	3 / 107	0.037
GO:0015718	monocarboxylic acid transport	3 / 107	0.038
GO:0015849	organic acid transport	3 / 107	0.041
GO:0048167	regulation of synaptic plasticity	3 / 107	0.042
GO:0035601	protein deacylation	3 / 107	0.044
GO:0098732	macromolecule deacylation	3 / 107	0.045

Table S12: List of the significant CpG-transcript pairs identified for the CpG sites associated with smoking (LC-AwS).

CpG ID	p-value	r	Transcript ID	Gene Name	Transcript	RefSeq ID
cg21566642	5.56E-23	-0.59	ILMN_306943	*LRRN3*	ILMN_1773650	NM_001099660.1
cg05575921	3.50E-23	-0.58	ILMN_174401	*LRRN3*	ILMN_2048591	NM_018334.3
cg05575921	2.35E-14	-0.46	ILMN_20796	*MGAT3*	ILMN_1853824	NM_002409.4
cg05575921	3.47E-14	-0.46	ILMN_162388	*MUC1*	ILMN_1756992	NM_001044391.1
cg05575921	1.31E-12	-0.43	ILMN_14069	*FUCA1*	ILMN_1752728	NM_000147.3
cg06126421	8.19E-12	-0.42	ILMN_20343	*SLC26A8*	ILMN_1755843	NM_052961.2
cg06126421	1.31E-10	0.40	ILMN_163755	*LFNG*	ILMN_1663080	NM_001040167.1
cg21566642	3.22E-10	-0.40	ILMN_308518	*IGSF9B*	ILMN_1660204	NM_014987.1
cg05575921	1.71E-10	-0.39	ILMN_7859	*PID1*	ILMN_1671891	NM_017933.3
cg05575921	1.82E-10	-0.39	ILMN_181709	*SASH1*	ILMN_2185984	NM_015278.3
cg06126421	3.30E-10	-0.39	ILMN_174093	*F5*	ILMN_1709233	NM_000130.4
cg05575921	3.21E-10	-0.39	ILMN_16567	*ASGR2*	ILMN_2342638	NM_080914.1
cg21566642	7.99E-10	0.39	ILMN_21945	*PTGDR*	ILMN_2062714	NM_000953.2
cg06126421	6.18E-10	-0.38	ILMN_6606	*CA4*	ILMN_1695157	NM_000717.2
cg06126421	6.20E-10	-0.38	ILMN_28738	*RGL4*	ILMN_1663422	NM_153615.1
cg06126421	6.31E-10	-0.38	ILMN_307349	*ENTPD1*	ILMN_1773125	NM_001098175.1
cg06126421	8.57E-10	-0.38	ILMN_18399	*TLR5*	ILMN_1722981	NM_003268.4
cg06126421	9.21E-10	-0.38	ILMN_17922	*FCAR*	ILMN_2379967	NM_133271.1
cg06126421	9.36E-10	-0.38	ILMN_5816	*RALB*	ILMN_1676358	NM_002881.2
cg05575921	8.50E-10	-0.38	ILMN_13048	*STAB1*	ILMN_1655987	NM_015136.2
cg05575921	9.94E-10	-0.38	ILMN_169862	*ALPL*	ILMN_1701603	NM_000478.3
cg06126421	1.21E-09	-0.38	ILMN_11644	*C16ORF57*	ILMN_1765880	NM_024598.2
cg06126421	1.28E-09	-0.38	ILMN_19989	*DGAT2*	ILMN_1681520	NM_032564.2
cg05575921	1.20E-09	-0.38	ILMN_16567	*ASGR2*	ILMN_1694966	NM_080914.1
cg06126421	1.45E-09	-0.38	ILMN_29514	*ADM*	ILMN_1708934	NM_001124.1
cg06126421	1.54E-09	-0.38	ILMN_28962	*BASP1*	ILMN_1651826	NM_006317.3
cg06126421	1.57E-09	-0.38	ILMN_11272	*C5ORF32*	ILMN_1761566	NM_032412.3
cg06126421	1.59E-09	-0.38	ILMN_16193	*DHRS13*	ILMN_1790781	NM_144683.3
cg05575921	1.43E-09	-0.38	ILMN_1879	*LOC153561*	ILMN_1767377	NM_207331.2
cg06126421	1.82E-09	-0.37	ILMN_3665	*NCF4*	ILMN_1785005	NM_000631.3
cg06126421	1.98E-09	-0.37	ILMN_28136	*MMP9*	ILMN_1796316	NM_004994.2
cg06126421	2.05E-09	-0.37	ILMN_26692	*MGAM*	ILMN_1714643	NM_004668.1
cg06126421	2.08E-09	-0.37	ILMN_18651	*GPR97*	ILMN_1765941	NM_170776.3
cg06126421	2.73E-09	-0.37	ILMN_164363	*KCNJ15*	ILMN_1675756	NM_170736.1
cg06126421	3.02E-09	-0.37	ILMN_164815	*OSM*	ILMN_1780546	NM_020530.3
cg06126421	4.43E-09	-0.37	ILMN_17438	*STX2*	ILMN_1726805	NM_194356.1
cg06126421	4.53E-09	-0.37	ILMN_15812	*SLC2A3*	ILMN_1775708	NM_006931.1
cg06126421	5.11E-09	0.36	ILMN_11424	*MATK*	ILMN_2319000	NM_139354.2
cg05575921	4.72E-09	-0.36	ILMN_19154	*FPR3*	ILMN_2203271	NM_002030.3
cg06126421	5.52E-09	-0.36	ILMN_34902	*LOC642684*	ILMN_1700855	XM_926137.1
cg21566642	9.63E-09	0.36	ILMN_20846	*WDR67*	ILMN_1744240	NM_145647.2
cg06126421	6.83E-09	-0.36	ILMN_181903	*C19ORF59*	ILMN_1762713	NM_174918.2
cg21566642	1.30E-08	-0.36	ILMN_26089	*PI16*	ILMN_1766264	NM_153370.2
cg21566642	1.45E-08	0.36	ILMN_25398	*FLJ14213*	ILMN_1697491	NM_024841.3
cg06126421	9.32E-09	-0.36	ILMN_33519	*LOC642103*	ILMN_1712999	XM_936233.1
cg05575921	8.77E-09	-0.36	ILMN_17337	*QSOX1*	ILMN_2411282	NM_001004128.2
cg06126421	1.06E-08	-0.36	ILMN_167573	*SIPA1L2*	ILMN_1732923	NM_020808.3
cg06126421	1.12E-08	-0.36	ILMN_25848	*SLC25A44*	ILMN_1810514	NM_014655.1
cg06126421	1.15E-08	-0.36	ILMN_23908	*SLC2A14*	ILMN_1668865	NM_153449.2
cg06126421	1.18E-08	-0.36	ILMN_15164	*AQP9*	ILMN_1715068	NM_020980.2
cg06126421	1.20E-08	-0.36	ILMN_756	*ZNF438*	ILMN_1678494	NM_182755.1
cg06126421	1.26E-08	-0.36	ILMN_18651	*GPR97*	ILMN_1798977	NM_170776.3
cg01940273	1.36E-08	-0.36	ILMN_349520	*LOC100132858*	ILMN_3247110	XM_001715131.1
cg06126421	1.40E-08	0.35	ILMN_27553	*RHOC*	ILMN_2313730	NM_175744.4
cg06126421	1.43E-08	-0.35	ILMN_14880	*NFIL3*	ILMN_1707312	NM_005384.2
cg05575921	1.26E-08	-0.35	ILMN_176497	*KIAA1881*	ILMN_1852022	XM_001130790.1

cg21161138	9.39E-09	-0.35	ILMN_165620	*TNFRSF13B*	ILMN_1759075	NM_012452.2
cg06126421	1.54E-08	-0.35	ILMN_25101	*DYSF*	ILMN_1810420	NM_003494.2
cg06126421	1.55E-08	-0.35	ILMN_19425	*SRPK1*	ILMN_1798804	NM_003137.3
cg06126421	1.67E-08	-0.35	ILMN_11581	*OPLAH*	ILMN_1711030	NM_017570.2
cg06126421	1.73E-08	-0.35	ILMN_18651	*GPR97*	ILMN_1815054	NM_170776.3
cg06126421	1.74E-08	-0.35	ILMN_10394	*SH3GLB1*	ILMN_1766045	NM_016009.2
cg06126421	1.78E-08	-0.35	ILMN_3601	*SLC22A4*	ILMN_1685057	NM_003059.2
cg05575921	1.57E-08	-0.35	ILMN_27475	*SLC9A8*	ILMN_1690625	NM_015266.1
cg06126421	1.98E-08	-0.35	ILMN_3665	*NCF4*	ILMN_2335704	NM_000631.3
cg06126421	2.89E-08	-0.35	ILMN_18817	*PGS1*	ILMN_2075051	NM_024419.3
cg06126421	2.92E-08	-0.35	ILMN_8722	*FCAR*	ILMN_2365091	NM_133280.1
cg06126421	3.05E-08	-0.35	ILMN_21850	*MAPK14*	ILMN_1737627	NM_001315.1
cg06126421	3.39E-08	-0.35	ILMN_167872	*SLC22A4*	ILMN_2050911	NM_003059.2
cg06126421	3.46E-08	-0.35	ILMN_26264	*PADI4*	ILMN_1807529	NM_012387.1
cg02451831	4.90E-08	-0.35	ILMN_124625	*HS.572444*	ILMN_1907834	
cg06126421	3.57E-08	-0.35	ILMN_4100	*LMNB1*	ILMN_2126706	NM_005573.2
cg06126421	3.65E-08	-0.34	ILMN_7658	*CASP4*	ILMN_1678454	NM_001225.3
cg01940273	3.90E-08	-0.34	ILMN_24176	*GFRA2*	ILMN_1656300	NM_001495.4
cg05575921	3.33E-08	-0.34	ILMN_38886	*PIM3*	ILMN_1707748	XM_938171.2
cg06126421	3.82E-08	-0.34	ILMN_5773	*B4GALT5*	ILMN_1685824	NM_004776.2
cg05575921	3.41E-08	-0.34	ILMN_71267	*HS.12513*	ILMN_1818677	
cg06126421	4.25E-08	-0.34	ILMN_165233	*ANTXR2*	ILMN_1812926	NM_058172.3
cg05575921	3.85E-08	0.34	ILMN_15995	*CD160*	ILMN_1742001	NM_007053.2
cg06126421	4.42E-08	-0.34	ILMN_12367	*ACSL1*	ILMN_1684585	NM_001995.2
cg06126421	4.56E-08	-0.34	ILMN_19321	*YIPF1*	ILMN_2052163	NM_018982.3
cg06126421	4.87E-08	-0.34	ILMN_16747	*LILRA5*	ILMN_1726545	NM_181879.1
cg06126421	5.51E-08	-0.34	ILMN_164215	*LOC728417*	ILMN_1665540	XM_001130364.1
cg02451831	8.02E-08	0.34	ILMN_21641	*EIF2B1*	ILMN_1753716	NM_001414.2
cg01940273	6.19E-08	0.34	ILMN_9867	*TCERG1*	ILMN_2398039	NM_006706.3
cg02451831	9.17E-08	0.34	ILMN_1506	*SEH1L*	ILMN_1708619	NM_001013437.1
cg06126421	6.91E-08	-0.34	ILMN_6510	*QPCT*	ILMN_1741727	NM_012413.3
cg05575921	6.18E-08	0.34	ILMN_19572	*PSME2*	ILMN_1786612	NM_002818.2
cg02451831	9.21E-08	0.34	ILMN_306788	*COMMD7*	ILMN_1810334	NM_001099339.1
cg06126421	7.58E-08	-0.34	ILMN_27578	*MTMR3*	ILMN_1803925	NM_021090.3
cg06126421	7.62E-08	0.34	ILMN_26701	*MXD4*	ILMN_1756541	NM_006454.2
cg05575921	6.76E-08	-0.34	ILMN_167544	*IGLL3*	ILMN_2083066	NM_001013618.1
cg06126421	7.66E-08	-0.34	ILMN_4895	*PCNX*	ILMN_1740010	NM_014982.2
cg05575921	6.81E-08	0.34	ILMN_8593	*CX3CR1*	ILMN_2088437	NM_001337.3
cg05575921	6.90E-08	0.34	ILMN_20313	*CYP4F22*	ILMN_1708303	NM_173483.2
cg06126421	8.40E-08	0.34	ILMN_27076	*CD247*	ILMN_1676924	NM_000734.2
cg06126421	8.44E-08	0.34	ILMN_27459	*DNAJC8*	ILMN_1698258	NM_014280.2
cg05575921	7.47E-08	-0.34	ILMN_20454	*SSPN*	ILMN_1775486	NM_005086.3
cg06126421	8.72E-08	-0.34	ILMN_28019	*PHF21A*	ILMN_1699496	NM_016621.2
cg06126421	8.76E-08	-0.34	ILMN_163517	*LIMK2*	ILMN_2367671	NM_016733.2
cg06126421	8.79E-08	-0.34	ILMN_19321	*YIPF1*	ILMN_1803564	NM_018982.3
cg05575921	7.88E-08	0.34	ILMN_8593	*CX3CR1*	ILMN_1745788	NM_001337.3
cg05575921	8.65E-08	-0.33	ILMN_163240	*FKBP9L*	ILMN_2089977	NM_182827.1
cg06126421	1.07E-07	-0.33	ILMN_10796	*TREM1*	ILMN_1688231	NM_018643.2
cg01940273	1.13E-07	-0.33	ILMN_363987	*WDFY4*	ILMN_3236551	NM_020945.1
cg05575921	1.02E-07	-0.33	ILMN_166018	*LOC441124*	ILMN_1778111	XM_499022.3

Table S13: Gene ontology categories significant in overrepresentation analysis of transcripts related to the CpGs associated with smoking (LC-AwS).

ID	Description	GeneRatio	p-value
GO:0009617	response to bacterium	10 / 79	0.000
GO:0003006	developmental process involved in reproduction	10 / 79	0.000
GO:0009611	response to wounding	9 / 79	0.004
GO:0006954	inflammatory response	9 / 79	0.004
GO:0055082	cellular chemical homeostasis	8 / 79	0.016
GO:0071345	cellular response to cytokine stimulus	8 / 79	0.025
GO:0031667	response to nutrient levels	7 / 79	0.002
GO:0009991	response to extracellular stimulus	7 / 79	0.004
GO:0098542	defense response to other organism	7 / 79	0.006
GO:0042060	wound healing	7 / 79	0.016
GO:0042742	defense response to bacterium	6 / 79	0.000
GO:0008643	carbohydrate transport	6 / 79	0.000
GO:0032496	response to lipopolysaccharide	6 / 79	0.005
GO:0002237	response to molecule of bacterial origin	6 / 79	0.007
GO:0071396	cellular response to lipid	6 / 79	0.046
GO:0015758	glucose transport	5 / 79	0.001
GO:0008645	hexose transport	5 / 79	0.001
GO:0015749	monosaccharide transport	5 / 79	0.001
GO:0042594	response to starvation	5 / 79	0.001
GO:0015711	organic anion transport	5 / 79	0.012
GO:0071496	cellular response to external stimulus	5 / 79	0.013
GO:0048608	reproductive structure development	5 / 79	0.035
GO:0061458	reproductive system development	5 / 79	0.036
GO:0001525	angiogenesis	5 / 79	0.040
GO:0006820	anion transport	5 / 79	0.040
GO:0046486	glycerolipid metabolic process	5 / 79	0.043
GO:0050829	defense response to Gram-negative bacterium	4 / 79	0.000
GO:0006639	acylglycerol metabolic process	4 / 79	0.002
GO:0006638	neutral lipid metabolic process	4 / 79	0.002
GO:0009267	cellular response to starvation	4 / 79	0.004
GO:0016241	regulation of macroautophagy	4 / 79	0.009
GO:0071222	cellular response to lipopolysaccharide	4 / 79	0.009
GO:0031669	cellular response to nutrient levels	4 / 79	0.011
GO:0071219	cellular response to molecule of bacterial origin	4 / 79	0.011
GO:0045765	regulation of angiogenesis	4 / 79	0.017
GO:0071216	cellular response to biotic stimulus	4 / 79	0.018

GO:0031668	cellular response to extracellular stimulus	4 / 79	0.019
GO:1901342	regulation of vasculature development	4 / 79	0.024
GO:0009266	response to temperature stimulus	4 / 79	0.028
GO:2000785	regulation of autophagosome assembly	3 / 79	0.001
GO:0044088	regulation of vacuole organization	3 / 79	0.003
GO:0006641	triglyceride metabolic process	3 / 79	0.009
GO:0032092	positive regulation of protein binding	3 / 79	0.009
GO:2000379	positive regulation of reactive oxygen species metabolic process	3 / 79	0.011
GO:0000045	autophagosome assembly	3 / 79	0.014
GO:1905037	autophagosome organization	3 / 79	0.016
GO:0010827	regulation of glucose transport	3 / 79	0.021
GO:0045766	positive regulation of angiogenesis	3 / 79	0.022
GO:1904018	positive regulation of vasculature development	3 / 79	0.031
GO:0038061	NIK/NF-kappaB signaling	3 / 79	0.037
GO:0000910	cytokinesis	3 / 79	0.038
GO:0051099	positive regulation of binding	3 / 79	0.040
GO:2000377	regulation of reactive oxygen species metabolic process	3 / 79	0.043
GO:1902115	regulation of organelle assembly	3 / 79	0.045
GO:2000045	regulation of G1/S transition of mitotic cell cycle	3 / 79	0.047

Figure S1: Boxplots for the 25 CpGs significantly different between cases and controls sorted according to increasing p-values from left to right. The two CpG sites not associated with smoking (LC-non-AwS) are indicated with green boxes.

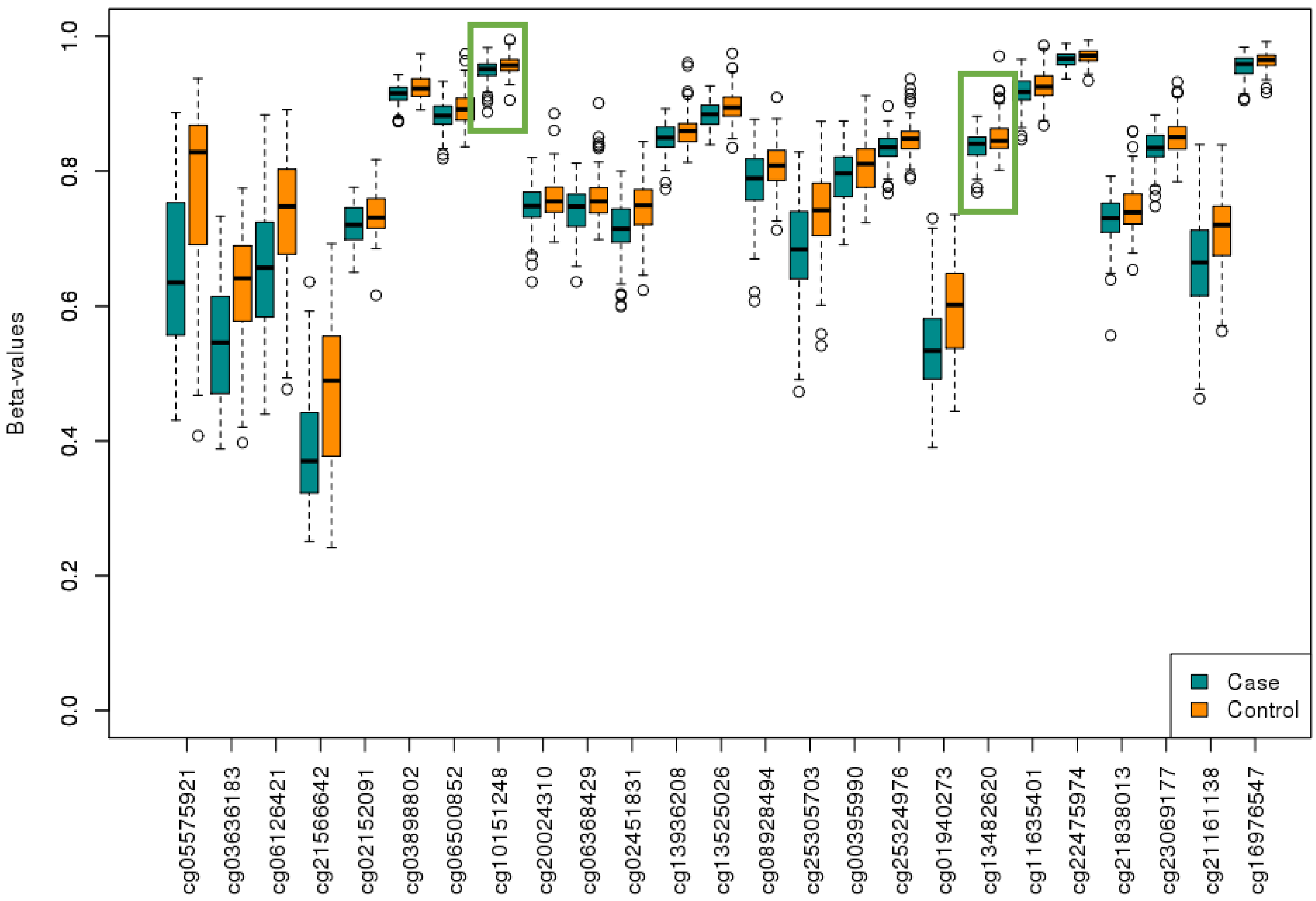

Figure S2: Volcano plot of the results of the unadjusted logistic regression models comparing methylation levels between cases and controls.

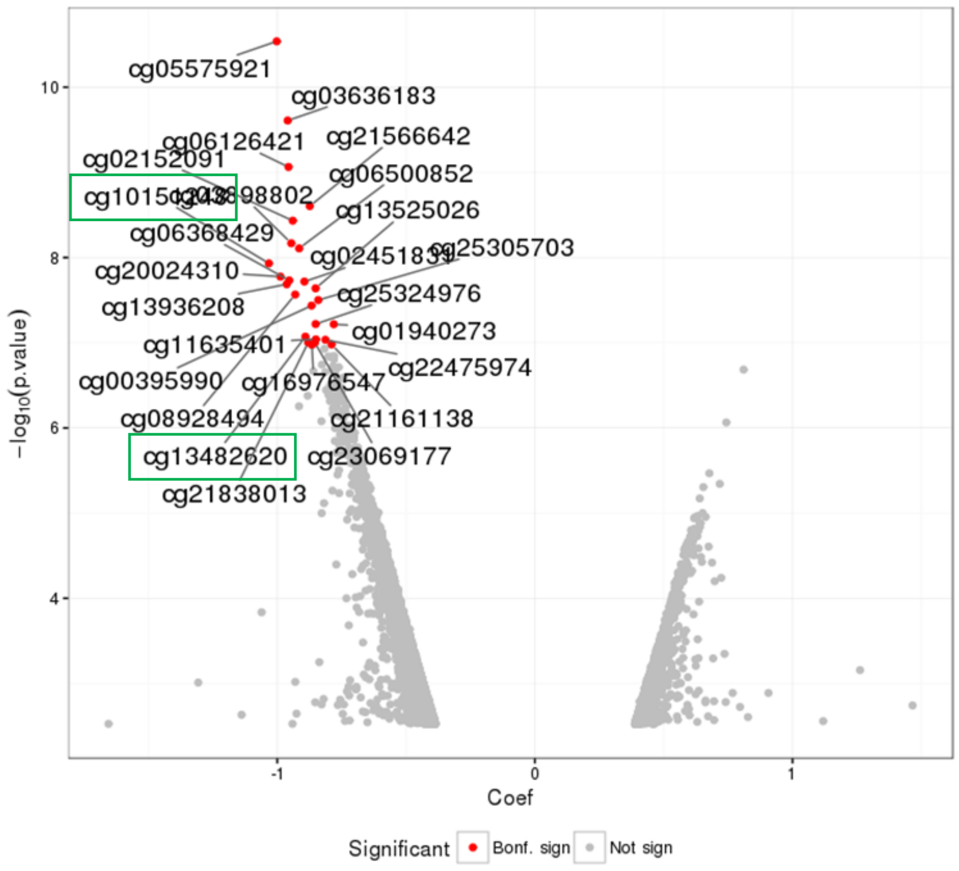

These models include denoised DNA methylation levels (DNA methylation adjusted for technical covariates and matching variables; see Method section) as an independent variable. The two CpG sites not associated with smoking (LC-non-AwS sites) are indicated with green boxes.

Figure S3: The p-value distribution of the unconditional logistic regression models comparing methylation levels between cases and controls and adjusted for different covariates.

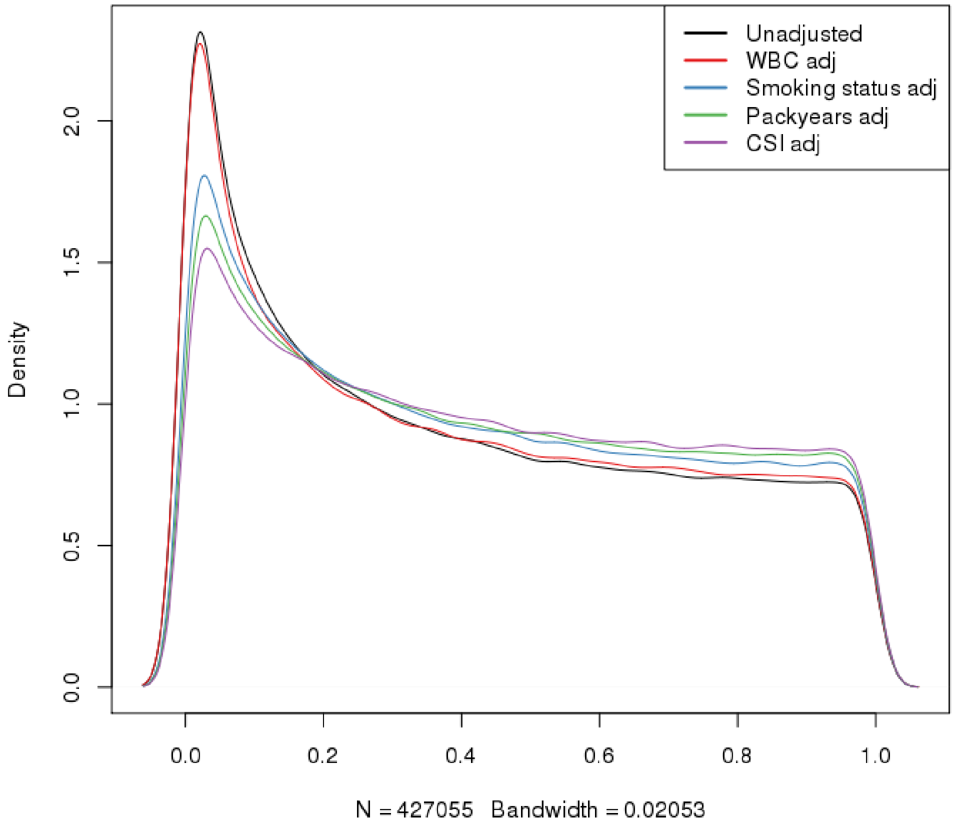

WBC: White Blood Cells, CSI: Comprehensive smoking index.
These models include denoised DNA methylation levels (DNA methylation adjusted for technical covariates and matching variables; see Method section) as an independent variable.

Figure S4: Heatmap of the correlation of the 25 candidate CpGs.

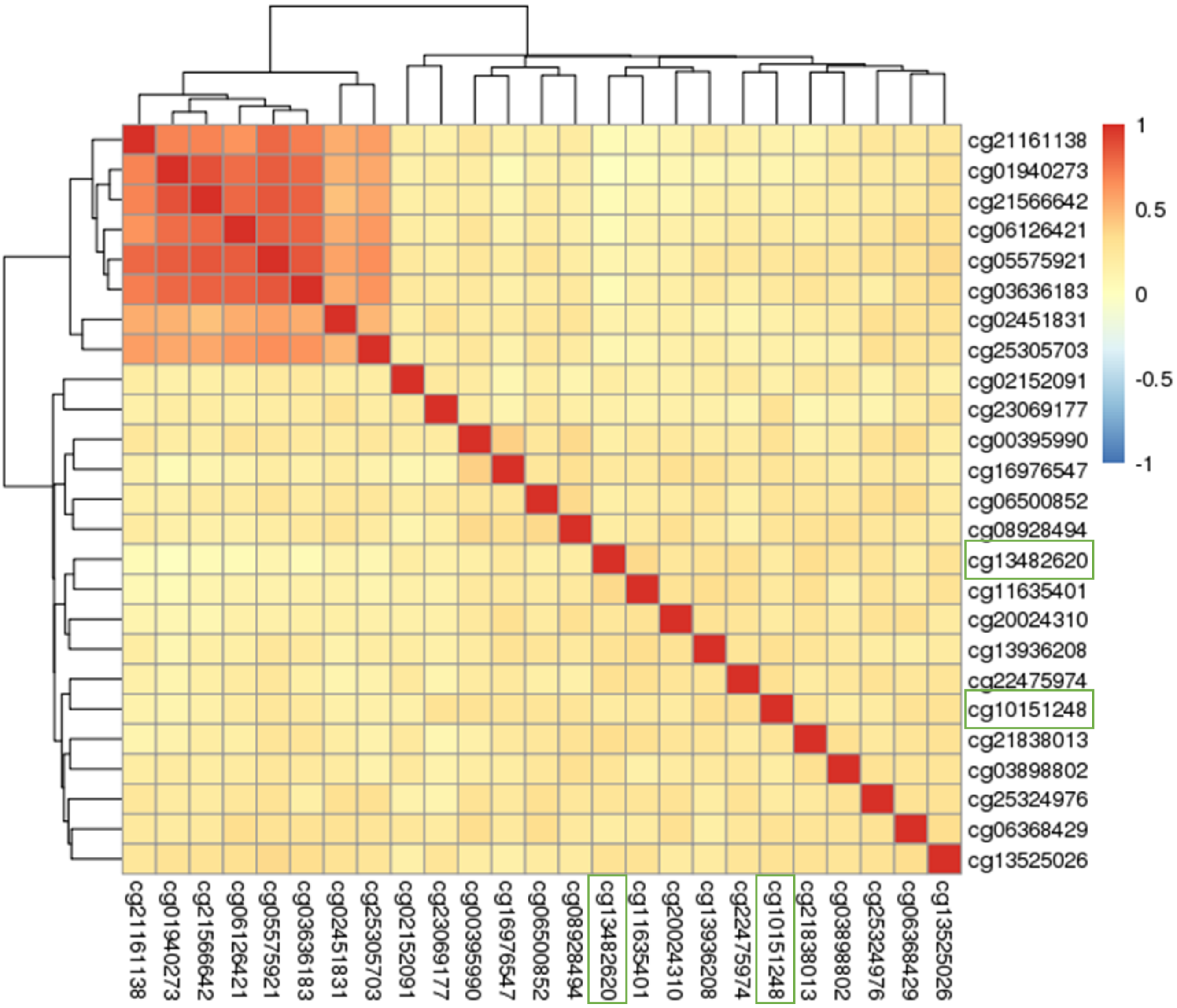

The two CpG sites not associated with smoking (LC-non-AwS sites) are indicated with green boxes

Figure S5: Heatmap of correlation for the 'second order' CpG and their associated transcripts.

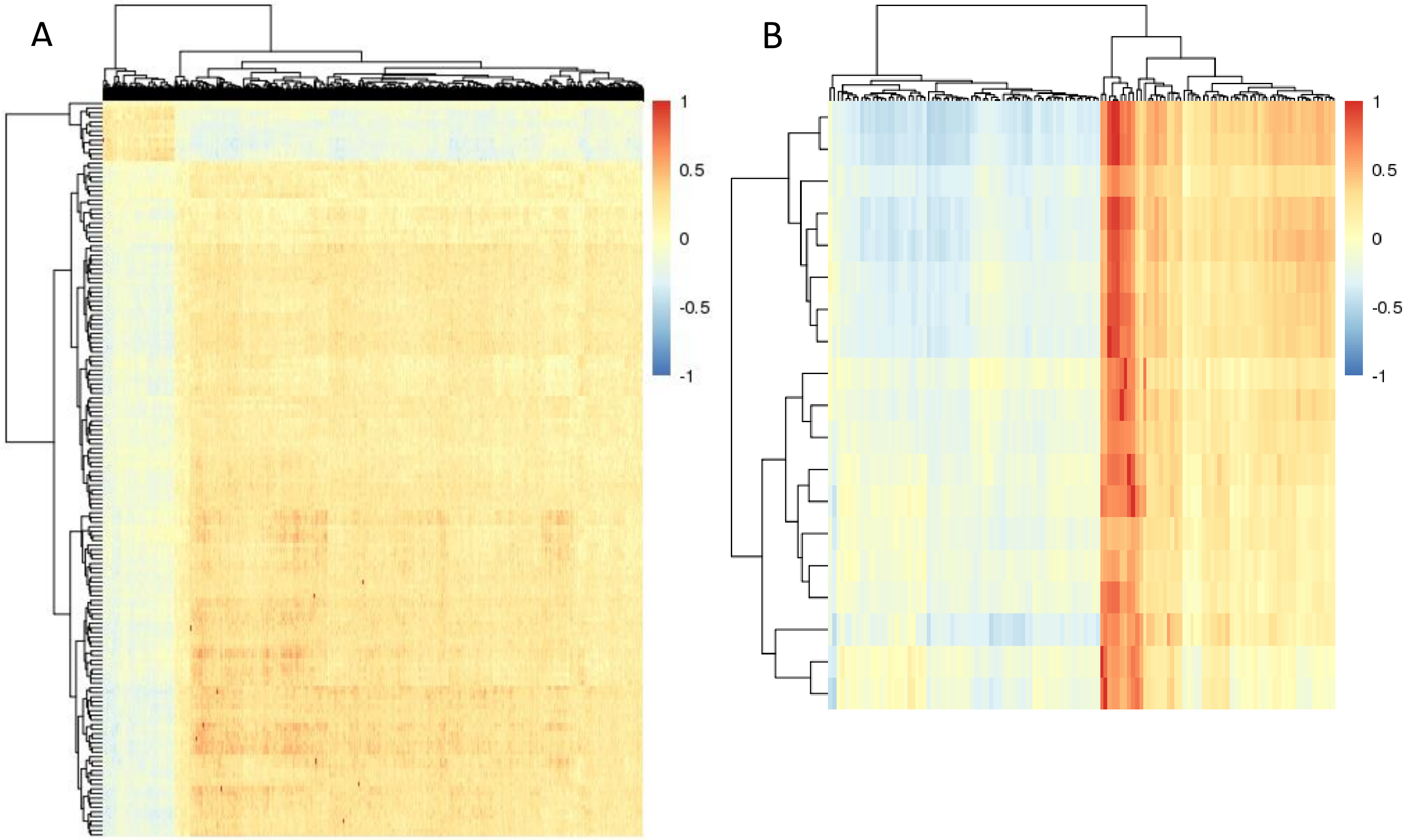

A) the 'denoised' methylation levels for the CpGs associated to the two CpG sites not associated with smoking (160 for cg10151248-*PC* and 1876 for cg13482620-*B3GNTL1*; and B), the 'denoised' expression levels for the transcripts associated with the n_1' 'second order' CpG associated to the CpG sites not associated with smoking (19 and 129 unique transcripts for CpGs associated to cg10151248-*PC* and cg13482620-*B3GNTL1*, respectively).
